# Disulfide‐Assisted Organic Polysulfide Cathode Design Enables Improved Kinetics in Lithium‐Sulfur Batteries

**DOI:** 10.1002/smsc.202500419

**Published:** 2025-11-18

**Authors:** Ruihua Li, Haoteng Wu, Haiwei Wu, Zhihua Lin, Frederik Bettels, Hairu Wei, Chong Wang, Wenhao Jia, Zhijian Li, Lin Zhang

**Affiliations:** ^1^ College of Bioresources Chemical and Materials Engineering Shaanxi University of Science and Technology Xi'an 710021 China; ^2^ Institute for Solid State Physics Leibniz University Hannover Appelstrasse 2 30167 Hannover Germany

**Keywords:** high‐sulfur loading, kinetics, lithium‐sulfur battery, organic sulfides, paper‐based electrode, tetramethylthiuram disulfide

## Abstract

Lithium‐sulfur batteries (LSBs) is fundamentally limited by the “shuttle effect” and poor kinetics. To address these challenges, this study proposes an approach through developing a novel organic polysulfide composite cathode with high sulfur loading. By implementing a radical reaction between elemental sulfur and a disulfide of tetramethylthiuram disulfide (TMTD), linear organic polysulfides (TMTD‐S) containing over 70 wt% sulfur are successfully synthesized. This kind of material features a covalently bonded R‐Sn‐R (R=C_2_H_6_N(S)) backbone. Further compounding with the conductive carbon (ECP600JD) and integrating into a paper‐based electrode help to improve the electrode's conductivity and optimized ion transport pathways. The obtained TMTD‐24S@ECP600JD cathode demonstrates a capacity retention rate of 79.1% after 250 cycles at 0.2C, far superior to traditional S@ECP600JD materials (14.1%). By increasing the sulfur content in TMTD, higher sulfur‐content linear organic polysulfides are also obtained. Among them, the TMTD‐54S@ECP600JD with 88 wt% sulfur content exhibits the best electrochemical performance and the highest lithium‐ion diffusion coefficient, delivering an initial discharge capacity of 941 mAh g^−1^ at 0.2C, with a capacity retention rate of 82.1% after 200 cycles. Even at a high rate of 2C, it still maintained a high specific capacity of 638.3 mAh g^−1^, making it a potential material for high‐performance Li‐S batteries.

## Graphical abstract

This study synthesizes linear organic polysulfides with sulfur content exceeding 70 wt% via a radical reaction between sulfur and tetramethylthiuram disulfide (TMTD). The material features a covalent R‐Sn‐R backbone, helps to suppress the shuttle effect and enhance kinetics.The TMTD‐S cathode demonstrates a high discharge capacity of 941 mAh g^‐1^ and good cycling performance.

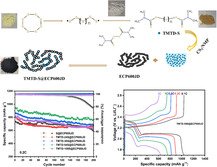

## 1 Introduction

Lithium‐sulfur batteries (LSBs) have emerged as a promising candidate for next‐generation high‐energy‐density storage systems, boasting a remarkable theoretical specific capacity of 1675 mAh g^−1^ and an exceptional energy density of 2600 Wh kg^−1^, substantially surpassing conventional lithium‐ion technologies.^[^ ^1^, ^2^, ^3^ ^]^ The inherent advantages of sulfur cathode—including natural abundance, environmental friendless, and cost‐effectiveness—further reinforce its potential for commercialization.^[^ ^4^, ^5^ ^]^ Nevertheless, three fundamental limitations persist in practical implementations: (1) progressive capacity fading during cycling, (2) low utilization of sulfur, and (3) restricted lifespan.^[^ ^6^ ^]^ These challenges are primarily associated with its poor kinetics and the dissolution and migration of lithium polysulfides (LiPSs) within the electrolyte, which significantly hinder discharging‐charging processes and contribute to active material depletion and lithium anode corrosion.^[^ ^7^, ^8^, ^9^, ^10^ ^]^

Substantial efforts have been dedicated to enhancing kinetics and mitigating LiPSs shuttling through diverse strategies: (1) separator modification to enhance battery kinetics,^[^ ^11^, ^12^ ^]^ (2) utilization of chemisorption and catalytic activity in polar materials to accelerate LiPS to Li_2_S conversion,^[^ ^13^ ^]^ (3) synchronous enhancement of sulfur utilization and reaction kinetics through physical confinement and catalytic interface design in composite electrodes,^[^ ^14^, ^15^ ^]^ (4) functional electrolyte additives,^[^ ^16^, ^17^ ^]^ and (5) electrocatalyst integration to accelerate LiPS conversion kinetics.^[^ ^18^, ^19^ ^]^ While these approaches have achieved significant success in enhancing reaction kinetics under conventional sulfur loadings, the persistent challenges of sluggish reaction kinetics and severe LiPS dissolution become particularly pronounced at high sulfur loadings, remaining predominant factors governing capacity degradation.^[^ ^20^ ^]^ Therefore, further research is critically needed to fundamentally control LiPS dissolution and enhance reaction kinetics, especially for high‐sulfur‐loading cathodes.^[^ ^21^, ^22^ ^]^

Former investigations reveal that the reversible cleavage/reformation of S‐S bonds constitutes the fundamental operating mechanism of sulfur cathode—a phenomenon not exclusive to elemental sulfur but ubiquitous in diverse sulfur‐containing compounds.^[^ ^23^, ^24^ ^]^ This understanding has catalyzed the development of non‐elemental sulfur cathodes featuring stabilized S‐S bond frameworks (e.g., organic polysulfides,^[^ ^25^ ^]^ metal polysulfides^[^ ^26^ ^]^), which enable direct reversible conversion between S_2_ and S_4_ short‐chain species during cycling, thereby circumventing the formation of long‐chain soluble LiPSs (Li_2_S_*x*_, *x* > 4). Among these, organic disulfide/trisulfide compounds have garnered particular attention due to their molecular design flexibility, enhanced cycle stability, and economic viability.^[^ ^27^, ^28^, ^29^ ^]^ Although early studies on organic disulfides/trisulfides (e.g., diphenyl disulfide) have demonstrated the concept of S‐S bond reversibility, these compounds are often plagued by limited capacity (due to short sulfur chains) and insufficient cycling stability.^[^ ^30^ ^]^ Moreover, recent strategies focusing on sulfur/nitrogen‐doped carbon (S/NC) composites primarily rely on the physical confinement and chemical adsorption of sulfur within a host matrix.^[^ ^31^, ^32^, ^33^, ^34^ ^]^ Therefore, achieving both high sulfur content and structural integrity remains a key scientific challenge.

Herein, we successfully synthesized linear organic sulfides with different sulfur contents through the addition reaction between sulfur and tetramethylthiuram disulfide (TMTD). The resulted tetramethylthiuram disulfide‐sulfur (TMTD‐S) exhibits molecular structure of R‐S*_n_*‐R (where R is C_2_H_6_N(S)), and the sulfur content exceeds 70 wt%. Since sulfur chains are covalently anchored in the organic molecular framework, long‐chain polysulfides cannot dissolve freely into the electrolyte during charge‐discharge cycles, thus avoiding the shuttle effect. In addition, bis(dimethylthiocarbamyl) sulfide (BDTS^Red^) forms soluble complexes (e.g., BDTS^Red^‐Li_2_S_6_) with polysulfides such as Li_2_S_6_ in the electrolyte, which promotes the local liquid‐phase transformation of polysulfides on the electrode surface and thereby enhances redox kinetics. To further improve its conductivity and cycling stability, TMTD‐S was composited with the high‐conductive carbon black of Ketjen Black (ECP600JD) to prepare the TMTD‐S@ECP600JD material. The TMTD‐S@ECP600JD material prepared through compositing not only maintains a high sulfur content but also optimizes the electrical conductivity of the electrode, improves cycling stability, and enhances the reversible discharge capacity. Specifically, TMTD‐54S@ECP600JD with a sulfur content of 88 wt% exhibits the best comprehensive performance, with a high lithium‐ion diffusion coefficient. Its initial discharge specific capacity at 0.2 C reaches 941 mAh g^−1^, and the capacity retention rate after 200 cycles can be 82.1%; even at a high rate of 2 C, it still shows a high specific capacity of 638.3 mAh g^−1^.

## 2 Result and Discussion

### 2.1 Synthesis and Characterization of TMTD‐S@ECP600JD Composites

**Figure 1:**
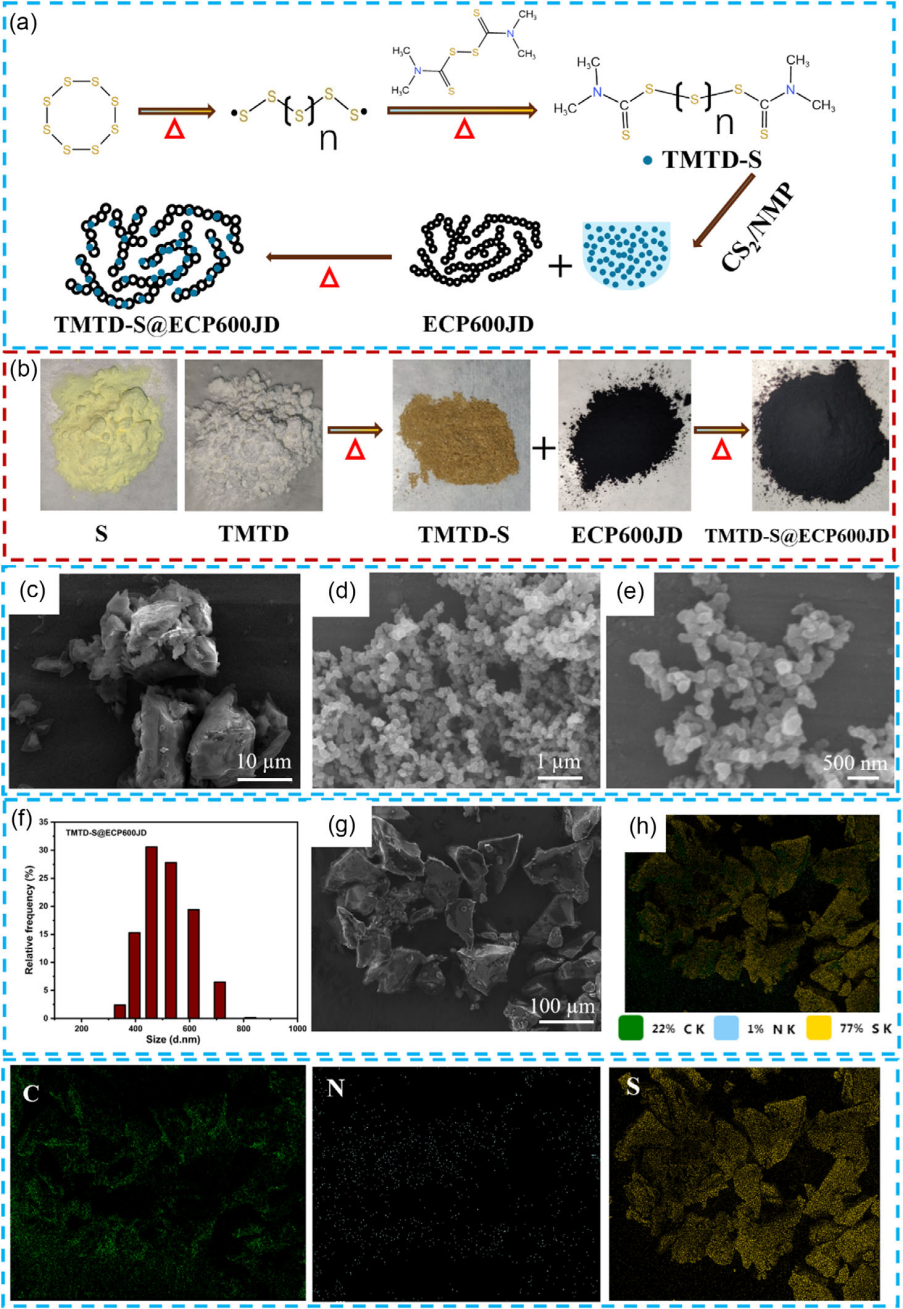
a) The reaction diagram of TMTD‐S synthesis. b) The image of color evolution during sample preparation. c–e) SEM images of TMTD‐S. f) The particle size distribution map of TMTD‐S@ECP600JD. g) SEM image of TMTD‐24S. h) The corresponding EDS spectra of C, S, and N elements.

TMTD‐S synthesis involves a free radical addition reaction where sulfur atoms selectively add to the thioether group of TMTD, which is similar to the formation of other organic sulfur‐based composites.^[^ ^35^ ^]^ The specific capacity of the organic sulfur cathode is positively correlated with the number of active sulfur atoms. Each sulfur atom contributes capacity through the transfer of two electrons during the discharge process. TMTD material is a common vulcanizing agent with a linear molecular structure and only one S‐S bond. Its theoretical capacity is relatively low. Through calculation, the theoretical specific capacity is only 223 mAh g^−1^, which limits its application in the field of batteries. Therefore, extending the length of the sulfur chain through in situ sulfidation of the sulfide group has become an effective strategy for increasing the specific capacity. When elemental sulfur and TMTD undergo pyrolysis in the molten state, the thioether bond undergoes homolytic cleavage to generate highly active TMTD radicals, while elemental sulfur dissociates to form sulfur radicals.^[^ ^36^ ^]^ During this process, the S radical can add to the TMTD radical, eventually forming TMTD‐S linear organic polysulfides with a long sulfur chain. As shown in **Figure** **1**a, S_8_ and TMTD were reacted via thermal melting to synthesize the linear organosulfur compound TMTD‐S. Subsequently, a solvent‐assisted dispersion strategy was used: TMTD‐S was dissolved in a CS_2_/NMP co‐solvent system (v/v = 1:1), uniformly blended with conductive substrate ECP600JD under ultrasonication, and finally consolidated to form the TMTD‐S@ECP600JD composite material. Furthermore, as can be seen from Figure 1b, the original sulfur powder (yellow) reacts with TMTD (grayish‐white) to form TMTD‐S with the characteristic yellowish‐brown color. This color‐change phenomenon provides an intuitive characterization basis for the successful reaction of sulfur with TMTD. The SEM image in Figure 1c shows that TMTD‐S exists in the form of irregular particles as high as tens of micrometers. After compounded with ECP600JD as shown in Figure 1d and Figure 1e, the prepared TMTD‐S@ECP600JD composite show a same morphology with nano‐sized ECP600JD, suggesting that TMTD‐24S is uniformly distributed on the ECP600JD surface. The particle size distribution in Figure 1f also indicates that the particles are mainly in the range of 500 nm, further suggesting the obtained TMTD‐24S@ECP600JD forms a composited cluster structure in which ECP600JD as substrate and TMTD‐24 can be easily melted and coated on it. Energy dispersion spectra (EDS) of the TMTD‐24S material are also shown in Figure 1g,h. The figure shows that C, N and S are uniformly distributed in different atomic percentages. The sulfur mass in the prepared TMTD‐24S material is 77%, which is very close to the theoretical calculated value of 76%. This result confirms the successful reaction of sulfur with TMTD at the elemental composition level. Furthermore, Figure S1, Supporting Information, shows that C and S are uniformly distributed in TMTD‐24S@ECP600JD in different atomic percentages, illustrating the uniform distribution of TMTD‐24S on the electrode.

**Figure 2:**
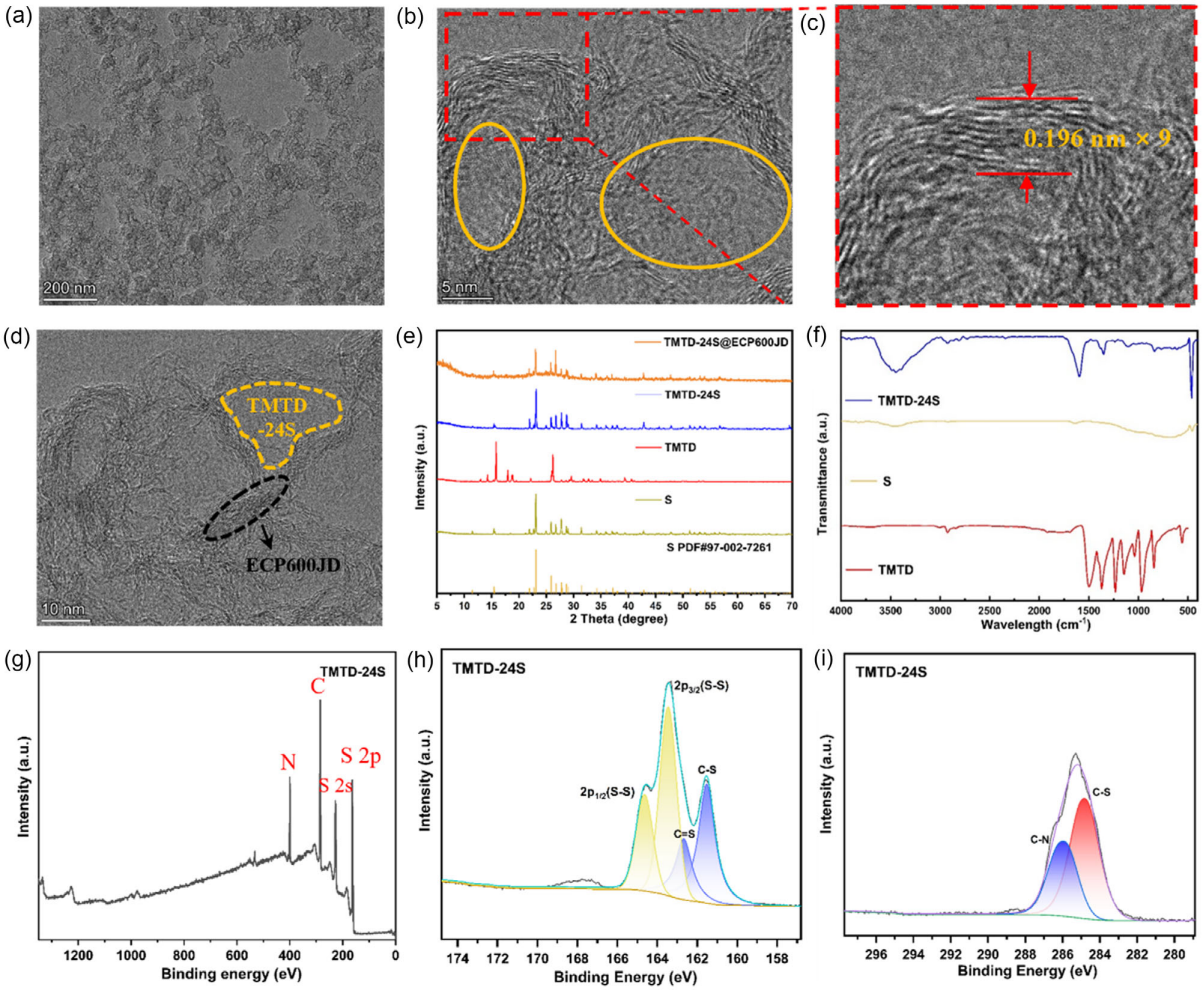
a–d) TEM images of TMTD‐24S at high resolution. e) XRD patterns of S, TMTD, TMTD‐24S and TMTD‐24S@ECP600JD. f) FT‐IR spectra of S, TMTD and TMTD‐24S. g) XPS full spectra of TMTD‐24S. h) S 2p XPS spectra of TMTD‐24S. i) C 1s XPS spectra of TMTD‐24S.

Furthermore, as can be seen from the TEM image in **Figure** **2**a, TMTD‐24S@ECP600JD composite presents a typical nanoparticle assembly structure. The high‐resolution TEM image in Figure 2b–d show that there are clear lattice fringes with a spacing of 0.196 nm, which is highly consistent with the standard crystal plane spacing of ECP600JD. The yellow marked area presents relatively blurred lattice fringes, which could be the signal of TMTD‐24S. This area simultaneously shows the coexistence phenomenon of amorphous phase and crystalline phase. The analysis of the X‐ray diffraction (XRD) pattern in Figure 2e shows the disappearance of the characteristic diffraction peaks of TMTD after the addition reaction, suggesting the cleavage process of its molecular structure. Subsequently, sulfur atoms are inserted to form TMTD‐24S composite, mainly showing a typical deceased crystallinity structure of S. After being composited with ECP600JD, the characteristic diffraction peaks of TMTD‐24S@ECP600JD are similar to those of TMTD‐24S and S, but both the peak intensity and peak width further decrease correspondingly. Fourier‐transform infrared (FTIR) spectroscopy further clarified the chemical structure changes of TMTD‐24S. As shown in Figure 2f, for the spectrum of TMTD, the absorption peaks at ≈1500 to 1601 cm^−1^ are attributed to the N‐C bond vibration, while the absorption peaks at 1038 to 1147 cm^−1^ correspond respectively to the C‐S and C(‐S)S bonds. Compared with TMTD, the intensity of the characteristic absorption peak of S‐S bonds at 497 cm^−1^ of TMTD‐24S was significantly increased, while the other peaks were relatively weakened. This is due to the introduction of S‐S bonds in the molecular chain. The newly formed S chain enhances the dipole moment variation of the S‐S bond through electron cloud rearrangement and simultaneously weakens the vibration of adjacent C‐S bonds.^[^ ^37^, ^38^ ^]^ Furthermore, the C‐N and C‐S peaks showed a redshift, while the C‐S peak at 971 cm^−1^ showed a blue shift. This can be attributed to the intermolecular conjugation effect caused by the insertion of new S chains.^[^ ^39^ ^]^ These changes further indicate that elemental sulfur has been successfully embedded into the TMTD structure with linear properties. The elemental composition and valence states of C, N, and S in TMTD and TMTD‐24S were systematically investigated using X‐ray photoelectron spectroscopy (XPS). As depicted in Figure 2g, the full‐spectrum scan of TMTD‐24S clearly reveals the presence of C, N, S 2s, and S 2p, which are consistent with the elemental spectra shown in Figure S2a, Supporting Information. This confirms the incorporation of carbon, nitrogen, and sulfur into the material. In Figure S2b, Supporting Information, the S 2p orbitals of TMTD were fitted into two distinct bipeaks, corresponding to C‐S bonds at 161.2 eV and 162.1 eV, and S‐S bonds at 163 eV and 164.1 eV. As illustrated in Figure 2h, compared to TMTD, TMTD‐24S exhibits a positive peak shift of 0.5 eV for the C‐S bond, resulting in binding energies of 161.7 eV and 162.6 eV. The S‐S bond peaks also experience shifts of 0.4 eV, reaching 163.4 eV and 164.5 eV. These observations indicate that the newly formed S chain induces intramolecular charge redistribution, causing the electron cloud of the C‐S bond to shift toward the sulfur‐rich region.

Additionally, the intensity of the S‐S splitting peak in TMTD‐24S has significantly increased, with the area ratio relative to the C‐S splitting peak changing from 1:1 (TMTD) to 2:1. These changes reflect an increase in the number of S‐S bonds within the material, corroborated by the FT‐IR data presented in Figure 2f, which further confirms the interaction between sulfur and TMTD. This suggests successful polymerization of sulfur with TMTD, enhancing the sulfur content in linear TMTD‐S. By comparing Figure 2i with Figure S3, Supporting Information, it is evident that the relative intensity and peak area ratios of the characteristic C‐N and C‐S bonds in TMTD and TMTD‐24S remain approximately constant at a ratio of 1:3, indicating stability in the number of C‐N and C‐S bonds within the thiuram molecular framework during the reaction process. Combined with the intensity change of the S‐S bond in the S 2p spectrum, it is further confirmed that sulfur mainly realizes polymerization with TMTD through the extension of the intermolecular S chain, without changing the thiuram‐based linear structure of TMTD.

**Figure 3:**
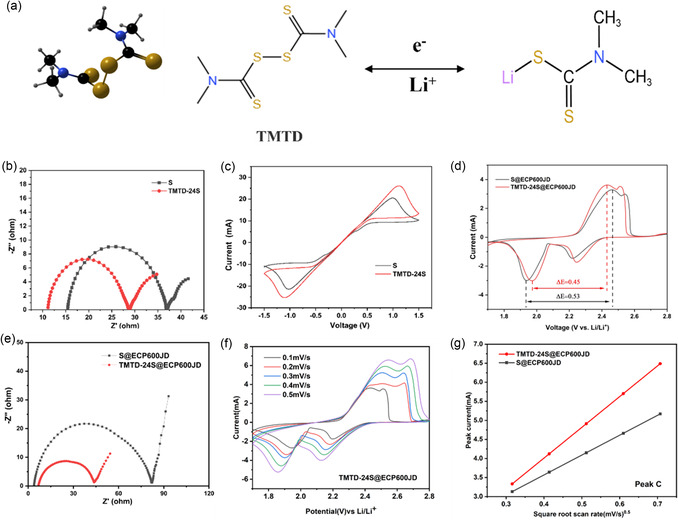
a) A schematic diagram of the redox reaction of TMTD during charging and discharging. b) EIS curves of symmetrical batteries. c) CV curves of symmetrical batteries at 1 mV s^−1^ scan rate. d) CV curves at 0.1 mV s^−1^ scan rate of S@ECP600JD and TMTD‐24S@ECP600JD and e) EIS curves. f) CV curves of TMTD‐24S@ECP600JD at scan rates from 0.1 to 0.5 mV s^−1^. CV peak current of g) peak C versus the square root of the scan rate.

In LSBs prepared with elemental sulfur, the insulating sulfur and lithium sulfide, as well as the shuttle effect induced by soluble LiPSs, severely limit the conversion kinetics and utilization rate of sulfur. How to achieve a rapid and reversible redox reaction is the key to realizing high‐performance LSBs. In order to reveal the electrochemical reaction process of linear organic sulfur, charge and discharge tests were first conducted on button batteries prepared with TMTD as the cathode. In Figure S4a, Supporting Information, the discharge specific capacity of TMTD at 0.05C is 209 mAh g^−1^, which is equivalent to 93.7% of the theoretical specific capacity (223 mAh g^−1^), demonstrating excellent utilization efficiency of the active material. Moreover, after 300 cycles at 1C, the discharge specific capacity is 115.1 mAh g^−1^, and the coulombic efficiency remains consistently above 99.5%. In Figure S4b, Supporting Information, the charge‐discharge process of TMTD exhibits the characteristic of a single plateau, which is highly consistent with the structural feature that the material contains only one S‐S bond. This single plateau confirms that its capacity contribution completely originates from the breaking and recombination of the S‐S bond, and the C‐S bond in the molecule does not participate in the redox reaction. Similar to former report,^[^ ^40^ ^]^ the discharge mechanism of TMTD is simulated as shown in **Figure** **3**a: during discharge, TMTD participates in the electrochemical reaction through the breaking of the S‐S bond and the lithiation reaction. Meanwhile, the N atoms with relatively high electronegativity in the molecular structure exhibit a certain electron delocalization effect, which helps to improve the electrical conductivity and electrochemistry of the material.

To further reveal the intrinsic regulatory mechanism of TMTD‐24S material on the redox reaction of polysulfides, a symmetrical battery system was constructed for testing. The internal resistance of a battery is intrinsically related to the reaction kinetics.^[^ ^41^ ^]^ The EIS spectra of the two materials were evaluated. As shown in Figure 3b, compared with pure sulfur, TMTD‐24S prepared by the composite of TMTD and sulfur has the smallest semicircle diameter in the high‐frequency region, indicating that it has a lower charge transfer resistance during the redox reaction. This may be because its linear organic sulfur structure provides a better charge transfer ability than pure sulfur, thereby accelerating the reaction. The electrochemical reaction process within the LSB was analyzed using CV curves. In the CV curve of Figure 3c, TMTD‐24S also exhibits a higher peak current response than pure sulfur, indicating that its redox reaction kinetics is faster.^[^ ^42^ ^]^ These results collectively reveal the advantages of TMTD‐24S in regulating the redox reaction kinetics.

In order to verify the reaction kinetics characteristics of the TMTD‐24S@ECP600JD battery, a CV test was carried out on the battery at a scanning rate of 0.1 mV s^−1^. As shown in Figure 3d, the CV curve of S@ECP600JD exhibits typical sulfur conversion characteristics. The reduction peaks at 2.35 V and 1.98 V correspond to the reduction of S_8_ to soluble LiPSs and the further reduction of LiPSs to solid Li_2_S_2_/Li_2_S, respectively; the broad oxidation peak at around 2.4 V corresponds to the oxidation of Li_2_S_2_/Li_2_S first to long‐chain LiPSs and then further oxidation back to S_8_. Compared with S@ECP600JD, the CV curve of TMTD‐24S@ECP600JD shows a narrower peak spacing (Δ*E*) and a stronger peak current response, which corresponds to the EIS curve of TMTD‐24S@ECP600JD in Figure 3e with low charge transfer impedance and high lithium‐ion diffusion rate. As shown in Figure 3f and Figure S5a, Supporting Information, during the discharge process, peaks A and B correspond to the reduction of S_8_ to soluble LiPSs and further reduction of LiPSs to solid Li_2_S_2_/Li_2_S, respectively. During the charging process, oxidation peaks C and D correspond to the reverse transformations: oxidation of Li_2_S_2_/Li_2_S to long‐chain LiPSs and further oxidation of LiPSs to S_8_. The reduction and oxidation peaks of TMTD‐24S@ECP600JD both exhibit the strongest current response. This observation indicates that TMTD‐24S@ECP600JD can effectively promote the rapid reversible conversion among sulfur, LiPSs, and Li_2_S_2_/Li_2_S in Li‐S batteries. On the other hand, the lithium‐ion diffusion coefficient was analyzed using the Randles‐Sevcik Equation (1)

(1) $$I_{p}=\left(2.69\times 10^{5}\right)n^{1.5}SD^{0.5}\Delta C_{\text{Li}}V^{0.5}$$

here, *I*_p_ represents the peak current, *n* is the number of electrons, *S* is the geometric area of the cathode, *D* is the diffusion coefficient of lithium ions, *V* is the scanning rate, and Δ*C*_Li_ is the change in Li^+^ concentration during the electrochemical reaction process. The peak values of the oxidation and reduction processes are selected to determine the diffusion coefficient. The slope of the curve (*I*_p_/*v* ^0.5^) is positively correlated with the lithium‐ion diffusion coefficient. The greater the slope of the curve, the higher the diffusion coefficient of lithium ions. As shown in Figure 3g and Figure S5b–d, Supporting Information, the TMTD‐24S@ECP600JD electrode exhibits a steeper slope than the S@ECP600JD electrode, indicating a faster lithium‐ion diffusion rate. To verify the improvement in electrode conductivity, a comparison was made between EIS curves of TMTD‐24S and TMTD‐24S@ECP600JD presented in Figure S6, Supporting Information. After compounding with the highly conductive carbon material ECP600JD, the charge transfer resistance of the TMTD‐24S@ECP600JD electrode was reduced by ≈5 times compared to that of the TMTD‐24S electrode. This significant reduction in resistance directly reflects a substantial improvement in the interfacial reaction kinetics between the electrode and electrolyte, which is attributed to the optimization of the overall electronic conduction network of the composite material and the enhancement of its charge transport capability.

**Figure 4:**
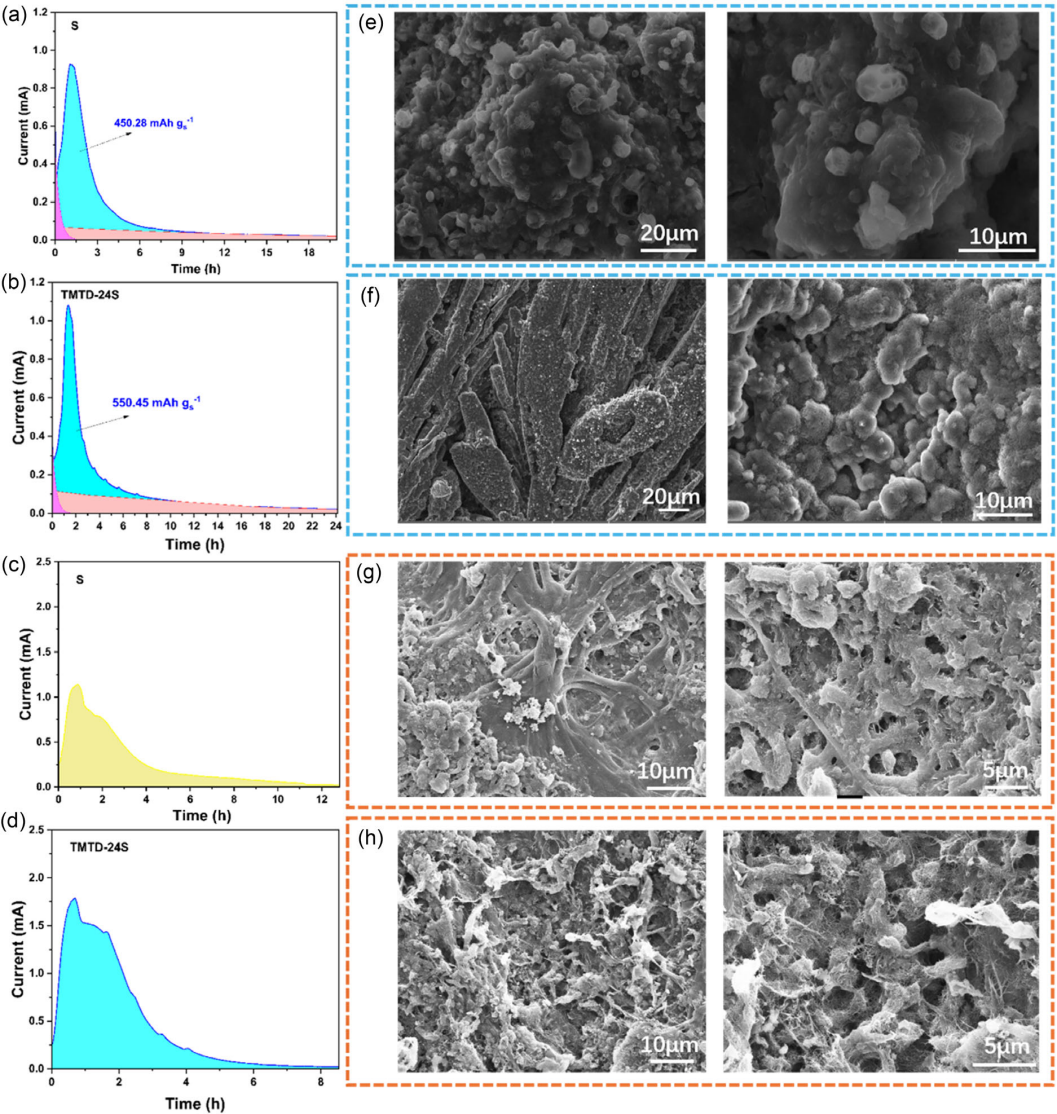
Li_2_S nucleation curves of a) S and b) TMTD‐24S on sulfur‐free paper. Li_2_S dissolution curves of c) S and d) TMTD‐24S. Corresponding electrode morphologies after Li_2_S nucleation on sulfur‐free paper of e) S and f) TMTD‐24S. Corresponding electrode morphologies after Li_2_S dissolution on sulfur‐free paper of g) S and h) TMTD‐24S.

The regulation mechanism of TMTD‐24S on the sulfur reduction pathway of LSBs was revealed through the potentiostatic deposition method.^[^ ^43^ ^]^ As shown in **Figure** **4**a,b, in the Li_2_S_8_ electrolyte system with a sulfur concentration of 4 mol L^−1^, the TMTD‐24S system exhibits a Li_2_S deposition capacity of 550.45 mAh g^−1^ on the sulfur‐free carbon paper, while the pure S system is only 450.28 mAh g^−1^. By promoting the nucleation and deposition of Li_2_S, TMTD‐24S can effectively regulate the sulfur reduction reaction pathway during the discharge process. This helps to reduce the shuttle loss of active substances by promoting the generation and rapid conversion behavior of polysulfides. Subsequently, through constant‐voltage charging, the deposited Li_2_S is dissolved from the sulfur‐free carbon paper. The dissolution curves are shown in Figure 4c,d. The dissolution current intensity and area of TMTD‐24S are stronger than those of pure S. The greater current intensity indicates that TMTD‐24S has higher oxidation reaction kinetics, which is beneficial to charge conversion. The larger dissolution area corresponds to a higher capacity, which indicates that more Li_2_S is successfully oxidized in the TMTD‐24S system. Combining the fact that TMTD‐24S has stronger current and a higher curve area during both the nucleation and dissolution of Li_2_S, this indicates that under the same sulfur concentration (S: 4 mol L^−1^), TMTD‐24S can achieve a higher sulfur utilization rate than pure S. In order to observe the morphological evolution of the solid‐state Li_2_S products, SEM characterization was carried out on the surface of the sulfur‐free carbon paper electrodes after the deposition and dissolution processes, respectively. As can be seen from Figure 4e, in the pure S system, Li_2_S is agglomerated at the micron level and deposited irregularly. Such large‐scale deposition may hinder the subsequent reactions and reduce the utilization rate of sulfur. However, in Figure 4f, Li_2_S is uniformly deposited on the paper fibers. This may be because the N‐containing groups on TMTD‐24S have better intermolecular forces with the hydroxyl groups of the fibers and the carbon chain structure, which can promote the directional deposition of Li_2_S on the fibers. This corresponds to the stronger deposition current of TMTD‐24S shown in Figure 4b. Subsequently, the state of the deposited Li_2_S during dissolution was further studied through a constant‐voltage charging process. As shown in Figure 4g, when charging at a constant voltage of 2.35 V, it can be observed that the solid deposition layer gradually dissolves, and the paper‐based fibers and Ketjen Black on the sulfur‐free carbon paper are re‐exposed. However, there is still some Li_2_S on the fibers of the pure S system that has not been oxidized and dissolved. This may be due to the large Li_2_S particles formed during the deposition stage, which cannot be completely oxidized during the charging process and thus form residues. In Figure 4h, compared with the pure S system, the Li_2_S deposition layer on the fibers of the TMTD‐24S system is basically dissolved, and the fiber surface restores its initial porous structure.

**Figure 5:**
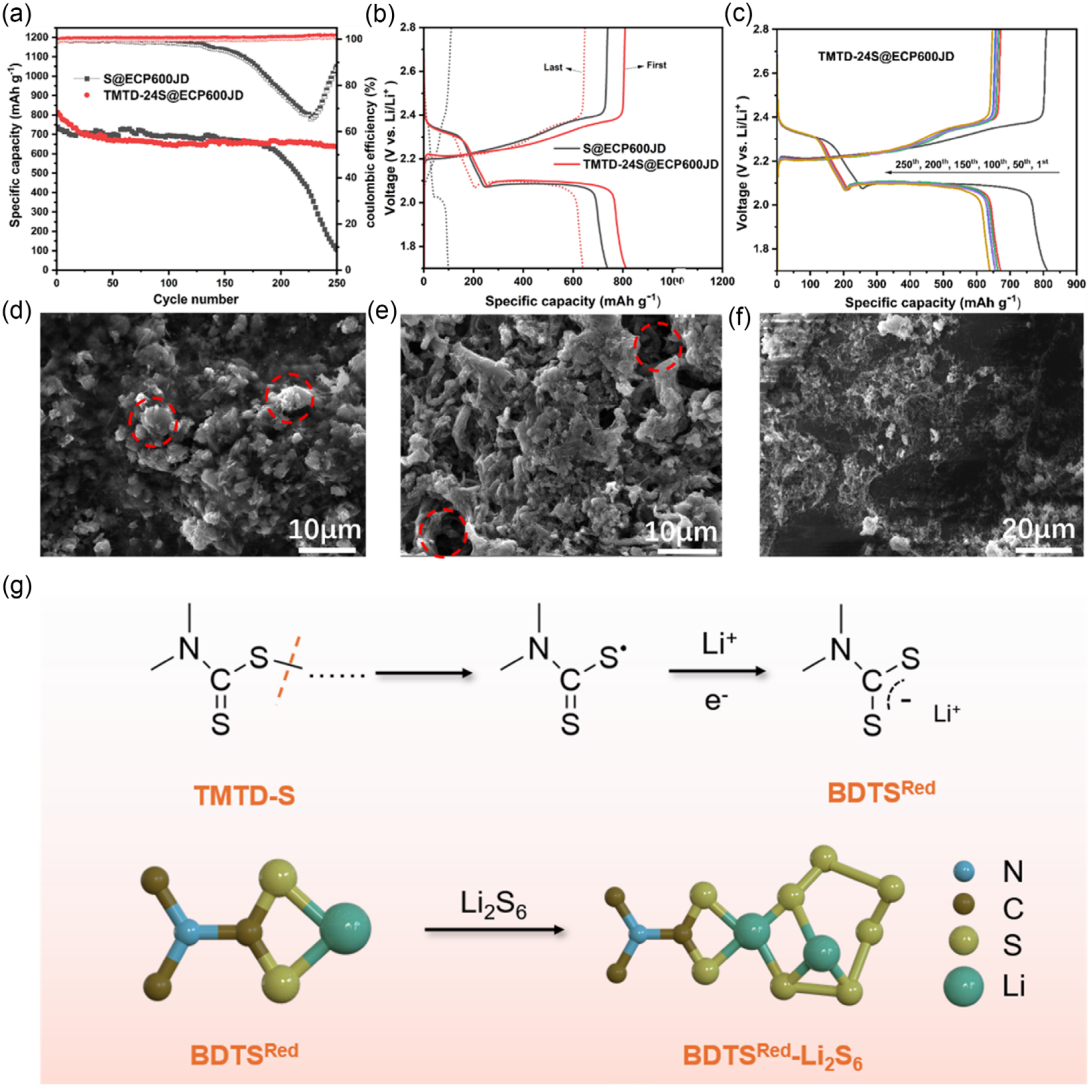
a) The cycle performance and coulombic efficiency curves of S@ECP600JD and TMTD‐24S@ECP600JD at 0.2 C. b) The charge‐discharge curves of the first and last cycles. c) The charge‐discharge curves of TMTD‐24S@ECP600JD at different cycles. SEM images of d) S@ECP600JD and e) TMTD‐24S@ECP600JD electrode before and after cycling. f) SEM images of TMTD‐24S@ECP600JD battery separator after cycling. g) Schematic illustration of TMTD‐S reduction mechanism and its complexation with Li_2_S_6_.

The long cycle performance curves of TMTD‐24S@ECP600JD and S@ECP600JD were compared and analyzed. The electrochemical cycling performance of TMTD‐24S@ECP600JD is shown in **Figure** **5**a. The initial discharge specific capacity is 807.4 mAh g^−1^ at 0.2C, and after 250 cycles, it still maintains a discharge specific capacity of 638.7 mAh g^−1^, with a capacity retention rate of 79.1%. The corresponding decay rate per cycle is only 0.084%. In contrast, the initial discharge specific capacity of S@ECP600JD is 739.1 mAh g^−1^, but it sharply decays to 104.2 mAh g^−1^ after 250 cycles, with a capacity retention rate of only 14.1% (as shown in Figure 5b). It is worth noting that the theoretical specific capacity (1326.5 mAh g^−1^) of TMTD‐24S@ECP600JD (S: 76 wt%) is calculated based on the theoretical specific capacity of sulfur (1675 mA g^−1^). In fact, its theoretical specific capacity is lower, but the discharge specific capacity exhibited by TMTD‐24S@ECP600JD (807.4 mAh g^−1^) is still higher than that of S@ECP600JD (739.1 mAh g^−1^), indicating that TMTD‐24S@ECP600JD has a higher utilization rate of active substances. By comparing and analyzing the long‐cycle performance curves of TMTD‐24S@ECP600JD and S@ECP600JD, it is found that both TMTD‐24S@ECP600JD and S@ECP600JD exhibit good stability in the initial stage of cycling (the first 150 cycles). This is mainly due to the synergistic effect brought about by the porous conductive network constructed by ECP600JD and the good dispersion of active substances. However, due to poor reduction and oxidation kinetics of elemental sulfur, long cycling of pure sulfur may lead to the passivation of the electrode because of continuing forming of unreversible of Li_2_S_2_/Li_2_S solids on surface. This not only weakens the fixing ability of the active substances but also causes LiPSs to be not transformed to Li_2_S_2_/Li_2_S s and easily dissolve out. These free LiPSs dissolve in the electrolyte and cause the shuttle effect, leading to irreversible loss of active substances and intensified side reactions with Li, and ultimately resulting in a rapid capacity decay (the capacity decay rate from 150 to 250 cycles is 80%). In contrast, TMTD‐24S@ECP600JD exhibits good cycling stability: the molecular skeleton of TMTD‐24S maintains excellent chemical stability during the charging and discharging processes, and its organic structure helps to improve the kinetics thus reducing the passivation of the electrode. The covalent bonds within the molecule restrict the sulfur chains within the molecular framework through chemical anchoring, significantly enhancing the solid‐liquid‐solid conversion reaction of LiPSs. By observing the discharge‐charge curves of TMTD‐24S@ECP600JD at different cycle numbers in Figure 5c and Figure S7, Supporting Information, it is fully demonstrated that it has low‐polarization charge/discharge plateaus and good cycling stability. The specific capacity decay from the 50th cycle (671.5 mAh g^−1^) to the 250th cycle (638.7 mAh g^−1^) is 32.8 mAh g^−1^. During 200 cycles, the average decay of TMTD‐24S@ECP600JD per cycle is 0.164 mAh g^−1^. This covalently fixed sulfur cathode material constructed through a molecular engineering strategy provides a new idea for solving the problem of capacity decay in LSBs and shows certain application prospects.

Through the study of the microstructural evolution of the cathodes of S@ECP600JD and TMTD‐24S@ECP600JD, the relationship between the electrode morphology and electrochemical performance can be revealed. The SEM morphologies before cycling are shown in Figure S8a,b and Figure S9a,b, Supporting Information. It can be seen that both cathodes exhibit a fibrous network structure on their surfaces. However, compared with S@ECP600JD, the cathode surface of TMTD‐24S@ECP600JD shows more obvious porous characteristics, which is conducive to the penetration of the electrolyte and the transport of lithium ions. This may be due to the appropriate particle size of TMTD‐24S@ECP600JD, which contains polar TMTD. Compared with S@ECP600JD, it has better dispersibility in water and thus is more conducive to uniform dispersion with fibers in the electrode. After the charge‐discharge cycle test, as observed in Figure S8c and S9c, Supporting Information, the surface structure of S@ECP600JD is significantly damaged, and many flaky thick passivation regions (red regions) appear in Figure 5d. The damage of this pore structure is due to the fact that after multiple cycles of sulfur, due to its poor oxidation kinetics, it continuously converts on the electrode surface to form a lithium sulfide passivation layer, and the pores on the electrode surface are gradually blocked and lose their activity. In contrast, in Figure 5e, the porous structure on the surface of TMTD‐24S@ECP600JD remains intact and there are decreased deposits after cycling, showing better structural stability. By comparing the changes in the microscopic morphology of the PP separator after cycling, direct visual evidence of the polysulfide shuttle effect is obtained. As shown in Figure S10, Supporting Information, a large number of passivation deposits appear on the separator of the S@ECP600JD battery, indicating that after multiple cycles of S@ECP600JD, due to the damage of the porous structure, LiPS tends to accumulate and deposit on the electrode‐separator interface. In Figure 5f, there are fewer deposits on the separator of the TMTD‐24S@ECP600JD battery. This also indirectly illustrates the excellent performance of the organic sulfur material of TMTD‐24S@ECP600JD in controlling the shuttle effect.

The weakly solvated electrolyte mediator bis(dimethylthiocarbamoyl) sulfide (BDTS) enables persistent quasisolid‐state polysulfide conversion by forming soluble BDTS^Red^‐Li_2_S_*x*_ complexes during cycling.^[^ ^44^ ^]^ Upon discharge initiation, BDTS reduces to BDTS^Red^ and complexes with surface Li_2_S_*x*_, facilitating localized liquid‐phase sulfur reduction near the electrodes. During charging, Li_2_S_*x*_ or S_8_ is oxidized while BDTS^Red^ is simultaneously reoxidized to BDTS, sustaining this dynamic mediation throughout the charge/discharge process.^[^ ^44^ ^]^ The TMTD selected in this paper has the same molecular structure as BDTS (only the number of sulfur atoms in the middle sulfur chain is different), so it can also undergo a complexation reaction with polysulfides such as Li_2_S_6_ during the discharge process. The proposed reduction mechanism of TMTD‐24S is shown in Figure 5g. When TMTD‐24S is discharged, the C‐S bond is broken to form the radical BDTS^Red^, and BDTS^Red^ forms a complex BDTS^Red^‐Li_2_S_6_ with Li_2_S_6_, promoting the rapid conversion of LiPSs, thereby suppressing the occurrence of the shuttle effect and improving the cycling stability.

### 2.2 The Material Characterization and Electrochemical Performance of TMTD‐S with Higher Sulfur Content

**Figure 6:**
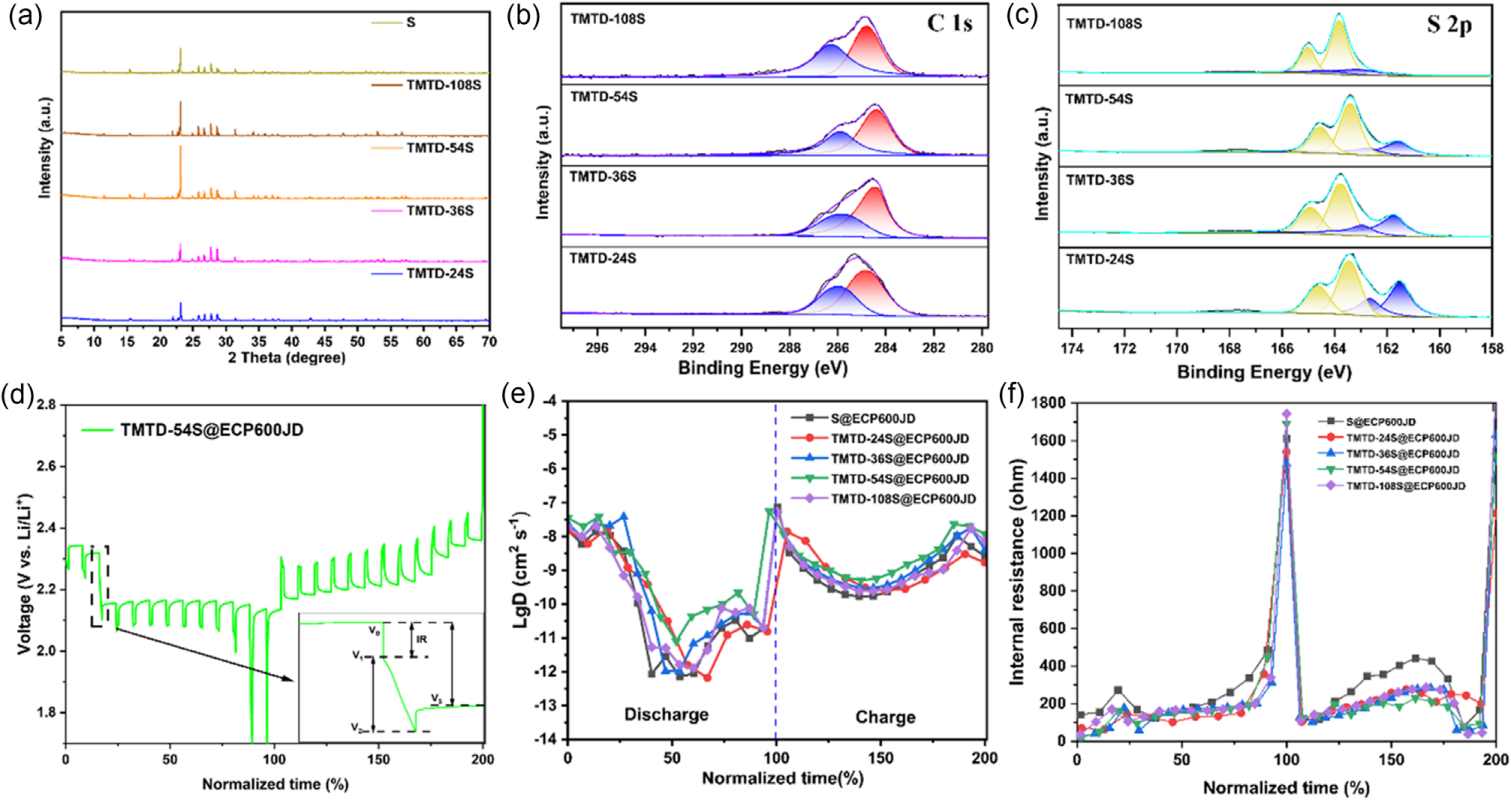
TMTD‐S a) XRD patterns with different sulfur contents, b) C 1s XPS spectra and c) S 2p XPS spectra. d) GITT curves and partial GITT curves of TMTD‐54S@ECP600JD electrodes. e) Lithium ion diffusion coefficient *D* curves of TMTD‐S@ECP600JD with different sulfur contents. f) Internal resistance curves.

TMTD‐24S shows advantages improving kinetics, but it still has deficiencies in terms of capacity performance. In order to obtain TMTD‐S materials with higher capacities, by increasing the proportion of sulfur in TMTD, TMTD‐36S (82 wt%), TMTD‐54S (88 wt%), and TMTD‐108S (94 wt%) with higher sulfur contents were synthesized. In the XRD patterns of TMTD‐S with different sulfur contents in **Figure** **6**a, as the sulfur content increases, the diffraction peak characteristics of sulfur are retained, indicating that sulfur still exists in the compound in the form of a crystalline state. The elemental sulfur crystallization peak observed in TMTD‐108S@ECP600JD is indicative of phase separation. This marks the material's partial transition from “covalently fixed sulfur” to “physically mixed sulfur”. As shown in Figure 6b, the relative intensities and relative areas of the C‐N bonds and C‐S bonds of TMTD‐S with different sulfur contents do not change significantly as the samples range from TMTD‐24S to TMTD‐108S, and the peak area is ≈1:3. With the increase of the sulfur content, the relative numbers of the C‐N bonds and C‐S bonds in the C 1s spectrum do not change. In the S 2p spectrum in Figure 6c, the relative intensities and relative areas of the S‐S bonds and C‐S bonds keep expanding, indicating that the number of S‐S bonds increases, which is consistent with the increase of the sulfur content. As shown in the TG‐DTG curve of Figure S11, Supporting Information, the sulfur content of the TMTD‐54S@ECP600JD electrode is 61.5%. Furthermore, in a nitrogen atmosphere, the TMTD‐54S@ECP600JD electrode exhibits no significant mass loss below 117.7 °C, and this negligible mass loss exerts no significant impact on the chemical structures of the active components (TMTD and S). This observation indicates that the electrode possesses relatively good thermal stability.

The reaction and diffusion kinetics of the material can be characterized more accurately through the galvanostatic intermittent titration technique (GITT).^[^ ^45^ ^]^ Since the GITT test requires observing the voltage change in the relaxation state after power off and the pulse time should be much shorter than the relaxation time, in order to ensure that the voltage during the relaxation process can be fully stabilized, an intermittent charge‐discharge strategy of constant current for 1 h and rest for 5 h is adopted, and the test is carried out at a small current of 0.2 mA cm^−2^. Figure 6d and Figure S12, Supporting Information, respectively display the GITT‐tested voltage curves of TMTD‐S@ECP600JD cathodes with different sulfur contents during a single charge‐discharge process. The lithium‐ion diffusion coefficient *D* of TMTD‐S@ECP600JD cathodes with different sulfur contents can be derived using formula (2).

(2) $$D\;=\frac{4}{\pi \tau }{\left(\frac{n_{m}V_{m}}{s}\right)}^{2}{\left[\frac{\Delta E_{s}}{\Delta E_{t}}\right]}^{2}$$

here, *τ* is the relaxation time, *n*_m_ is the molar number (mol, *V*_m_ is the molar volume (cm^3^ mol^−1^), *S* is the electrode/electrolyte contact area (cm^2^), Δ*E*_s_ is the total voltage change caused by the pulse (V), and Δ*E*_t_ is the voltage change of constant current charge/discharge (V). As depicted in Figure 6e, TMTD‐54S@ECP600JD exhibits the highest *D* value during the charging and discharging processes. This reveals that, among all the materials, the lithium‐ion diffusion coefficient of TMTD‐54S@ECP600JD, which has a sulfur content of 88%, is the greatest throughout the charge‐discharge cycle. However, TMTD‐108S@ECP600JD exhibits deteriorated ion transport properties due to phase separation and disruption of the conductive network. Evidently, lithium ions migrate at a swifter rate within the TMTD‐54S@ECP600JD cathode material. The efficient ion‐transport capability implies that the internal resistance for lithium‐ion transmission within the electrode is lower. This can markedly mitigate the polarization effect of the electrochemical reaction, thereby facilitating more rapid charging and discharging processes.^[^ ^46^ ^]^ Through the voltage variations of the TMTD‐S@ECP600JD electrodes with different sulfur contents during the intermittent titration process, the alterations in internal resistance during the test can be computed. As presented in Figure 6d, *V*_0_ denotes the equilibrium voltage of the final relaxation, *V*_1_ represents the potential at the commencement of the pulse, *V*_2_ is the potential at the conclusion of the pulse, and *V*_3_ is the equilibrium voltage of current relaxation.^[^ ^47^ ^]^ By leveraging these voltage changes and in conjunction with formula (3).

(3) $$\Delta R=\left|\Delta E_{s}-\Delta E_{t}\right|/I_{p}\;$$

here, Δ*R* represents the internal resistance of the battery (Ω), Δ*E*_s_ represents the potential difference between the two equilibrium voltages (Δ*E*_s_ = |*V*_3_−*V*_0_|), Δ*E*_t_ represents the potential difference between the start and end of charge and discharge (Δ*E*_s_ = |*V*_2_−*V*_1_|), *I*_p_ represents the current applied during constant current charging and discharging.

The variation curve of the battery's internal resistance as shown in Figure 6f can be obtained through formula (3). It can be seen that both during charging and discharging, all TMTD‐S@ECP600JD electrodes have relatively smaller internal resistance than the LSBs assembled with S@ECP600JD composite electrodes, which indicates that TMTD‐S can accelerate the diffusion of Li^+^ and improve the conversion efficiency of S during charging and discharging. It can promote the kinetics of redox reactions to a certain extent.

**Figure 7:**
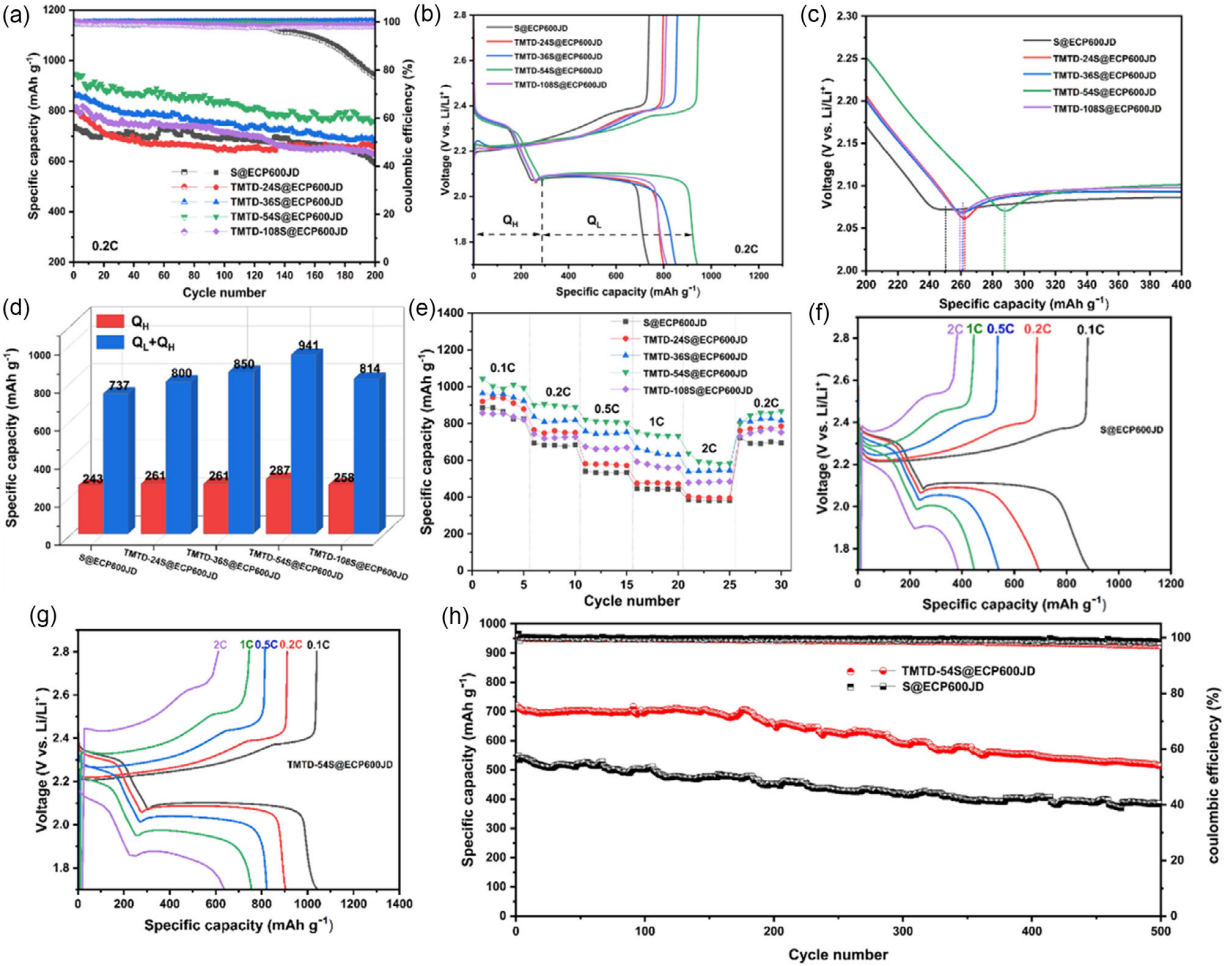
a) The cycle performance of Li‐S batteries assembled with S@ECP600JD, TMTD‐24S@ECP600JD, TMTD‐36S@ECP600JD, TMTD‐54S@ECP600JD, and TMTD‐108S@ECP600JD at 0.2 C. b) The charge‐discharge curves of the first cycle. c) The demarcation point of the high discharge stage (*Q*_H_) and the low discharge stage (*Q*_L_) at 0.2 C and d) the discharge specific capacity. e) The rate performance of different Li‐S batteries. The charge‐discharge curves of f) S@ECP600JD and g) TMTD‐54S@ECP600JD at different currents. h) The cycle performance of TMTD‐54S@ECP600JD and S@ECP600JD at 1C.

In order to explore the optimal ratio with the best electrochemical performance, the charge‐discharge performances of different organic sulfur cathode materials were also tested. As shown in **Figure** **7**a,b, under the conditions of an active material (TMTD‐S) loading of 2 mg cm^−2^ (the content of active materials in the electrode is 60 wt%) and 0.2 C, TMTD‐54S@ECP600JD exhibited the highest discharge specific capacity, which could reach 941 mAh g^−1^ in the first discharge cycle, far greater than that of TMTD‐36S@ECP600JD (850 mAh g^−1^), TMTD‐108S@ECP600JD (814 mAh g^−1^), TMTD‐24S@ECP600JD (800 mAh g^−1^), and S@ECP600JD (737 mAh g^−1^). Through a systematic analysis combined with the data in Table S1, Supporting Information, a nonlinear characteristic between the sulfur content of the organic sulfur material and its performance was found: when the sulfur content increased from 76 wt% (TMTD‐24S@ECP600JD) to 88 wt% (TMTD‐54S@ECP600JD), the discharge specific capacity showed an increasing trend. After the sulfur content exceeded 88 wt%, the performance of the composite material changed significantly: the discharge specific capacity of TMTD‐108S@ECP600JD (sulfur content 92 wt%) decreased. Analysis indicates that TMTD‐54S@ECP600JD corresponds to the optimal equilibrium point under the current material system. While achieving maximum incorporation of active sulfur, it does not induce severe phase separation or deterioration of electrical conductivity—while maintaining the high efficiency of the covalent sulfur‐fixation mechanism. However, excessive sulfur deviates from the original molecular design principles, causing the material to partially degrade into traditional sulfur/carbon composites, thereby triggering issues such as the Shuttle Effect and electrochemical kinetic hysteresis. Thus, the optimal molar ratio with the best electrochemical performance was determined to be 1:54 (TMTD‐54S@ECP600JD). It is worth noting that even under the condition of a low TMTD doping amount, the specific capacity of TMTD‐108S@ECP600JD was still higher than that of the pure sulfur system (S@ECP600JD). This confirms that embedding sulfur into the molecular chain of TMTD can effectively reconstruct the microscopic morphology and structure of sulfur, thereby increasing the utilization rate of active sulfur. The cycle stability test further verified the advantages of the TMTD‐S structure: TMTD‐54S@ECP600JD still had a discharge specific capacity of 772.4 mAh g^−1^ at 0.2C after 200 cycles, with a capacity retention rate of 82.1%, and the columbic efficiency remained above 99%. The typical discharge curve of a LSB is characterized by two clearly distinguishable operating potential intervals: the high‐voltage plateau (≈2.3–2.4 V) corresponds to the reduction of sulfur to long‐chain lithium polysulfide (Li_2_S_*x*_, 4 ≤ *x* ≤ 8), while the low‐voltage plateau (≈2.1 V) corresponds to the phase transition process of further reducing the long‐chain polysulfides to short‐chain Li_2_S_2_/Li_2_S. Among them, *Q*_H_ and *Q*_L_ respectively represent the capacity contributions of the two discharge stages. *Q*_H_ corresponds to the capacity of the high‐voltage stage, and *Q*_L_ corresponds to the capacity of the low‐voltage stage. The *Q*_L_/*Q*_H_ ratio, as a key performance indicator, directly reflects the effective utilization rate of the sulfur cathode material. The higher the *Q*_L_/*Q*_H_ value, the more sulfur has undergone the deep conversion process of being completely reduced to Li_2_S.^[^ ^48^ ^]^ As shown in Figure 7c,d, the *Q*_H_ and *Q*_L_ values of S@ECP600JD, TMTD‐24S@ECP600JD, TMTD‐36S@ECP600JD, TMTD‐54S@ECP600JD, and TMTD‐108S@ECP600JD are presented. Through calculation, the corresponding *Q*_L_/*Q*_H_ values are shown in Table S1, Supporting Information. Compared with S@ECP600JD (2.03), the composite linear organic sulfur material systems of TMTD‐S@ECP600JD all exhibited higher *Q*_L_/*Q*_H_ ratios, indicating that after introducing the organic structure, the conversion rate of sulfur was increased. Among them, TMTD‐54S@ECP600JD had the highest *Q*_L_/*Q*_H_ value (2.28), which indicates that TMTD‐54S@ECP600JD has a higher sulfur utilization rate, which is also consistent with its highest capacity. Figure 7e is the rate diagram of materials with different sulfur contents in the range from 0.1 C to 2 C, and Figure 7f,g and Figure S13, Supporting Information, are the corresponding charge‐discharge curves. It can be found that TMTD‐24S@ECP600JD, TMTD‐36S@ECP600JD, and TMTD‐54S@ECP600JD all exhibited higher discharge specific capacities than S@ECP600JD at all rates. It is worth noting that although the specific capacity of TMTD‐108S@ECP600JD (853 mAh g^−1^) at a low rate of 0.1 C was basically the same as that of S@ECP600JD (865 mAh g^−1^), when the rate increased to 0.2 C, 0.5 C, 1 C and 2 C, its capacity change was significantly higher than that of S@ECP600JD. All the TMTD‐modified materials exhibited excellent rate stability, and TMTD‐54S@ECP600JD performed particularly outstanding—after experiencing charge‐discharge cycles at a large current of 2 C, it could still exhibit a high specific capacity of 593.7 mAh g^−1^, which was 53.9% higher than that of S@ECP600JD under the same conditions (385.6 mAh g^−1^). This excellent rate performance can be attributed to the fact that the TMTD‐S material controls sulfur within the organic structure through molecular bonds, improving the electrode reaction kinetics, and thus maintaining stable electrochemical performance even at high current densities. As shown in Figure 7h, TMTD‐54S@ECP600JD also exhibited good cycle stability at a high rate of 1C. Combined with Figure S14, Supporting Information, the specific capacity at 1C was 720 mAh g^−1^. And after 500 cycles, it could still exhibit a discharge specific capacity of 513 mAh g^−1^, with a capacity decay rate of only 0.054% per cycle. These properties are precisely attributed to unique covalent sulfur fixation method: the molecular skeleton chemical fixation of sulfur chains in TMTD‐S. Figure S15, Supporting Information, presents the cycling and rate performance of the TMTD‐54S@ECP600JD battery under the conditions of a high sulfur loading of 4.6 mg cm^−2^ and a lean electrolyte with a ratio of 4.8 μL mg^−1^. As observed from the figure, the specific capacity exhibits a significant decline at higher current densities. This phenomenon can be attributed to the fact that, under high sulfur loading and lean electrolyte conditions, the electrochemical performance of the battery is mainly limited by the electrode reaction kinetic constraints and the intrinsic properties of the electrolyte itself. In addition, self‐discharge tests were also conducted. As shown in Figure S16, Supporting Information, the battery with TMTD‐54S@ECP600JD as the positive electrode was left to stand in an open circuit state for 7 days before the discharge capacity test was conducted. The discharge specific capacity after standing for 7 days was still as high as 936 mAh g^−1^, the capacity retention rate reached an astonishing 99.5%, and the capacity attenuation was negligible. This outstanding self‐discharge performance provides the most direct evidence that the TMTD‐S material effectively fixes sulfur species through its covalent bond molecular skeleton, greatly inhibiting the dissolution of polysulfides. As shown in Table S2, Supporting Information, compared with the state‐of‐the‐art organic sulfur cathode materials previously reported, the TMTD‐54S@ECP600JD composite developed in this work exhibits excellent overall electrochemical performance. While maintaining an extremely high sulfur content (88 wt%), it delivers a high reversible capacity and an extremely low cyclic decay rate. Its performance metrics are superior to or comparable with those of most analogous materials. Considering the comprehensive advantages of the TMTD‐54S@ECP600JD material in alleviating the shuttle of polysulfides, improving the stability of the electrode structure, and enhancing the reaction kinetics, it is expected to be a potential cathode material for high‐performance LSBs.

## 3 Conclusion

In this study, linear organic sulfides were used as substitutes for elemental sulfur, in which sulfur was fixed through chemical bonds to reduce intermediate dissolution and promote accelerated redox kinetics. Through the addition reaction between sulfur and TMTD, linear organic sulfide TMTD‐S with a high sulfur content (over 70 wt%) was successfully synthesized. This material has a framework of R‐S*_n_*‐R (where R is C_2_H_6_N(S)), and sulfur atoms are embedded in the molecular chain through covalent bonds, effectively suppressing the dissolution of polysulfides. During the charging and discharging processes, the C‐S bond in TMTD‐S breaks to form the radical BDTS^Red^, and the radical BDTS^Red^ forms a complex BDTS^Red^‐Li_2_S_6_ with Li_2_S_6_. This enhances the electrochemical activity and reaction kinetics of lithium polysulfides and alleviates the shuttle effect. Furthermore, when TMTD‐S is composited with ECP600JD to prepare TMTD‐S@ECP600JD, the electronic conductivity is significantly improved, and the ion transport path is optimized. Electrochemical tests show that after 250 cycles at a rate of 0.2 C, the capacity retention rate of TMTD‐24S@ECP600JD reaches 79.1%, far superior to the 14.1% capacity retention rate of the traditional S@ECP600JD material. In addition, the optimal feeding ratio of S to TMTD was determined to be 1:54. The TMTD‐54S@ECP600JD material (S:88 wt%) prepared with this feeding ratio exhibits the best electrochemical performance and the highest lithium‐ion diffusion coefficient. At a rate of 0.2 C, the initial discharge specific capacity reaches 941 mAh g^−1^, and after 200 cycles, 82.1% of the discharge specific capacity is retained. Even at a high rate of 2 C, it still has a high specific capacity of 638.3 mAh g^−1^. Therefore, TMTD‐54S@ECP600JD has excellent comprehensive performance and is a promising ideal cathode material for high‐performance LSBs.

## 4 Experimental Section

4.1

4.1.1

##### Synthesis of Linear Organosulfur Compounds

A mixture of 240 mg TMTD (1 mmol) and 768 mg sublimed sulfur (24 mmol) was thoroughly ground and transferred into an autoclave. The reaction was conducted under an argon atmosphere at 155 °C for 12 h, yielding a yellowish‐brown product designated as TMTD‐24S (S content: 76 wt%). By adjusting the molar ratio of sulfur to TMTD, organosulfur compounds with varying sulfur contents were synthesized: TMTD‐36S (S: 82 wt%), TMTD‐54S (S: 88 wt%), and TMTD‐108S (S: 94 wt%).

##### Preparation of Organosulfur/Carbon Composites

Ten milliliters of solvent was prepared by mixing 5 mL CS_2_ and 5 mL N‐methyl‐2‐pyrrolidone (NMP) in a 20 mL glass vial. Then, 1 g of TMTD‐24S was added to the solvent and stirred for 2 h until complete dissolution. Subsequently, 334 mg of carbon black of Ketjen Black (ECP600JD) was added, and the mixture was sonicated for 10 min. The solution was allowed to stand in a fume hood for 1 h to evaporate CS_2_, followed by vacuum drying at 80 °C for 12 h to remove residual NMP. The resulting solid powder was thermally fused under an argon atmosphere at 155 °C for 12 h to obtain TMTD‐24S@ECP600JD. Other linear organic sulfides (each 1 g) were synthesized into linear organic sulfur‐carbon composite materials using the same method.

##### Fabrication of Organosulfur‐Based Cathodes

Thirty milligrams of TMTD‐24S@ECP600JD and 15 mg of sodium dodecylbenzene sulfonate (SDBS) were dispersed in 15 mL deionized water under continuous stirring (500 rpm) for 1 h.

Separately, 3.5 mg of dried softwood fiber (SWF), 250 mg of 0.8 wt% bacterial nanocellulose dispersion (BNF), and 50 mg of 1 wt% carboxylated nanocellulose fiber gel (CNF) were added to 15 mL deionized water and stirred until to be totally mixed. Subsequently, 5 mg of carbon nanotubes (CNTs) and 10 mg of SDBS were added, and the mixture was sonicated for 3 min.

The sonicated dispersion from Step 2 was mixed with the sulfur dispersion from Step 1. The combined mixture was stirred to form a homogeneous slurry. The slurry was vacuum‐filtered through a 0.45 μm membrane, and the resulting electrode film was dried at 60 °C under −0.1 MPa for 12 h to obtain the TMTD‐24S@ECP600JD cathode. The loading of the active material of the electrode prepared by this method is ≈2 mg cm^−2^. S@ECP600JD electrode and other linear organic sulfur positive electrode with similar sulfur loading are also prepared by the above method.

##### Assembly of Cells

The assembly of lithium‐sulfur cells and symmetrical cells has been displayed in the supporting information.

##### Material Characterization

Scanning electron microscope (SEM) (Hitachi S‐4800) was used to observe the surface morphology. The lattice striations and plane spacing of the sample materials were observed using transmission electron microscopy (TEM) (FEI/Tecnai). The chemical composition of the electrode was analyzed using XPS (Al Kα, Kratos AXIS SUPRA^+^). XRD (Bruker/D8 Advance) was used to determine the crystal structure. FTIR spectra (Bruker VECTOR 22/N) were collected to analyze chemical bonding information.

##### Electrochemical Testing

CR2016 coin‐type Li‐S batteries were fabricated by assembling the prepared TMTD‐S based sulfur cathode, lithium metal anode, commercial polypropylene (PP) separator, and a 1 M LiTFSI‐DME/DOL electrolyte with 2 wt% LiNO_3_. Galvanostatic charge‐discharge (GCD) tests, GITT tests, and lithium psulfide deposition tests were performed on the batteries using battery testers (NEWARE CT4008T). The cyclic voltammetry (CV) curves were obtained by electrochemical workstation (CHI760E) at the specified scan rate within a voltage of 1.7 V–2.8 V. The electrochemical impedance spectroscopy (EIS) tests were implemented within a frequency of 0.01 Hz–100 kHz.

## Supporting Information

Supporting Information is available from the Wiley Online Library or from the author.

## Conflict of Interest

The authors declare no conflict of interest.

## Supporting information

Supplementary Material

## Data Availability

The data that support the findings of this study are available from the corresponding author upon reasonable request.
