# Supplementary material for: Disulfide‐Assisted Organic Polysulfide Cathode Design Enables Improved Kinetics in Lithium‐Sulfur Batteries
Source: Small Sci. 2025 Nov 18;6(1):e202500419. doi: 10.1002/smsc.202500419 (PMC12798778; doi:10.1002/smsc.202500419)
Supplement: Supplementary file 1 — Supplementary Material [file SMSC-6-e202500419-s001.pdf]

Supporting Information

Disulfides Assisted Organic Polysulfide Cathode Design Enables Improved Kinetics in Lithium-Sulfur Batteries

Ruihua Li[a, 1], Haoteng Wu[a, 1], Haiwei Wu[a, b,*], Zhihua Lin[b], Frederik Bettels[b], Hairu Wei[b], Chong Wang[a], Wenhao Jia[a], Zhijian Li[a, *], Lin Zhang[b, *]

[a]College of Bioresources Chemical and Materials Engineering, Shaanxi University of Science &Technology, Xi'an 710021. China

[b]Institute for Solid State Physics, Leibniz University Hannover, Appelstrasse 2, Hannover 30167, Germany

[1] Ruihua Li and Haoteng Wu contributed equally to this work

*Corresponding author. *E-mail address*: haiweiwufly@163.com (H Wu); wuhaiwei@sust.edu.cn ; zjli@sust.edu.cn (Z Li); l.zhang@fkp.uni-hannover.de (L Zhang)

Experimental Section

Chemicals. Sublimed sulfur (S_8, >99.9%) and carbon black of Ketjen Black (ECP600JD, >98%) were purchased from Zhongke Jinyan Technology Co., Ltd. (Beijing, China). Tetramethylthiuram disulfide (TMTD, >97%) was purchased from Shanghai Aladdin Technology Co., Ltd. Sodium dodecyl benzene sulfonate (SDBS, 36~38%) was purchased from Tianjin Damao Chemical Reagent Factory (Tianjin, China). Dust-free Paper Fiber (APF) was purchased from Kimberly-Clark Co., Ltd. (Shanghai, China). Carboxylated nanocellulose fiber gel (CNF, 1.0 wt %) and bacterial cellulose dispersion (BNF, 0.8 wt %) were purchased from Guilin Qihong Technology Co., Ltd. (Guangxi, China).

Assembly of lithium-sulfur batteries. Under argon atmosphere, the prepared TMTD-24S@ECP600JD positive electrode plate was cut into a 12 mm diameter wafer and used as the positive electrode of the battery. The negative electrode was a lithium wafer. Celgard 2500 microporous polypropylene (PP) film was used as the battery separator. The TMTD-24S@ECP600JD positive electrode plate was placed in the center of the positive shell, 0.03 mL of 1M LiTFSI-DME/DOL electrolyte with 2 wt% $LiNO_3$ was dropped onto the positive electrode material, the diaphragm was quickly covered on the positive electrode material to ensure that the diaphragm was fully soaked, and the lithium sheet was placed in the center of the negative shell. 0.01 mL of 1M LiTFSI-DME/DOL electrolyte with 2 wt% $LiNO_3$ was dropped onto the lithium sheet, the negative shell was attached to the positive shell, and the battery assembly was put into the hydraulic button battery sealer for sealing.

Preparation of lithium polysulfide solutions. Under argon atmosphere, 0.672 g of TMTD-24S and 0.092 g of Li_2S were weighed into a vial. Then, 1 M LiTFSI-DME/DOL commercial electrolyte solution containing 2 wt% $LiNO_3$ was added, and the mixture was diluted to 4 mL. The vial containing the solution was placed in an 80°C oil bath and stirred for 48 h until the solid powder completely dissolved, yielding a 0.17 M TMTD-24S solution. A blank control solution of 0.5 M Li_2S_8 was prepared by the same method using 0.092 g Li_2S and 0.56 g sublimed sulfur.

Assembly of Symmetric Cells. Symmetric cells were constructed using CR2016 coin cells in an argon-filled glovebox. Two sulfur-free carbon paper electrodes (12 mm diameter) were employed as both cathode and anode, with Celgard 2500 PP as the separator. 30 μL of 0.17 M TMTD-24S and 0.5 M Li_2S_6 (prepared from 0.092 g Li_2S and 0.4 g sublimed sulfur) solutions were added to the respective electrodes. The CV curves of symmetric cells were performed within the voltage range of -1.5–1.5 V (vs. Li^+/Li). EIS was tested by Chenhua Electrochemical Workstation (CHI760E) with a frequency ranging from 0.01 Hz to 100 kHz.

Supporting Tables

Tab.S1. The sulfur content and Q_L / Q_H value of S@ECP600JD, TMTD-24S@ECP600JD, TMTD-36S@ECP600JD, TMTD-54S@ECP600JD and TMTD-108S@ECP600JD.

	**TMTD-24S @ECP600JD**	**TMTD-36S @ECP600JD**	**TMTD-54S @ECP600JD**	**TMTD-108S @ECP600JD**	**S @ECP600JD**
**S（wt%）**	**74**	**82**	**88**	**94**	**100**
**Q_L/Q_H**	**2.06**	**2.25**	**2.28**	**2.15**	**2.03**

Tab.S2. Comparison of the electrochemical performance of our material with previously reported counterparts.

**Materials**	**Sulfur content (wt%)**	**Current density**	**Initial specific capacity (mAh g^{-1})**	**Cycle performance (Capacity retention rate)**	**References**
TMTD-54S @ECP600JD	88	0.2C	941	82.1% (200cycles)	**This Work**
Poly-S-UCO@SC	70	0.05C	93S6	42.3%(40cycles)	[1]
SPAN	53.6	0.2C	829	≈70%(100cycles)	[2]
DIXPS	64.6	0.1C	628	74%(1000cycles)	[3]
CoS_2/SPAN/CNT	43.2	0.2C	1799	48.9%(400cycles)	[4]
BP-SPAN	43	0.1C	2036	46.6%(200cycles)	[5]
MoS_2-NG@S-SH	70	0.5C	916	74.4%(200cycles)	[6]

Supporting figures

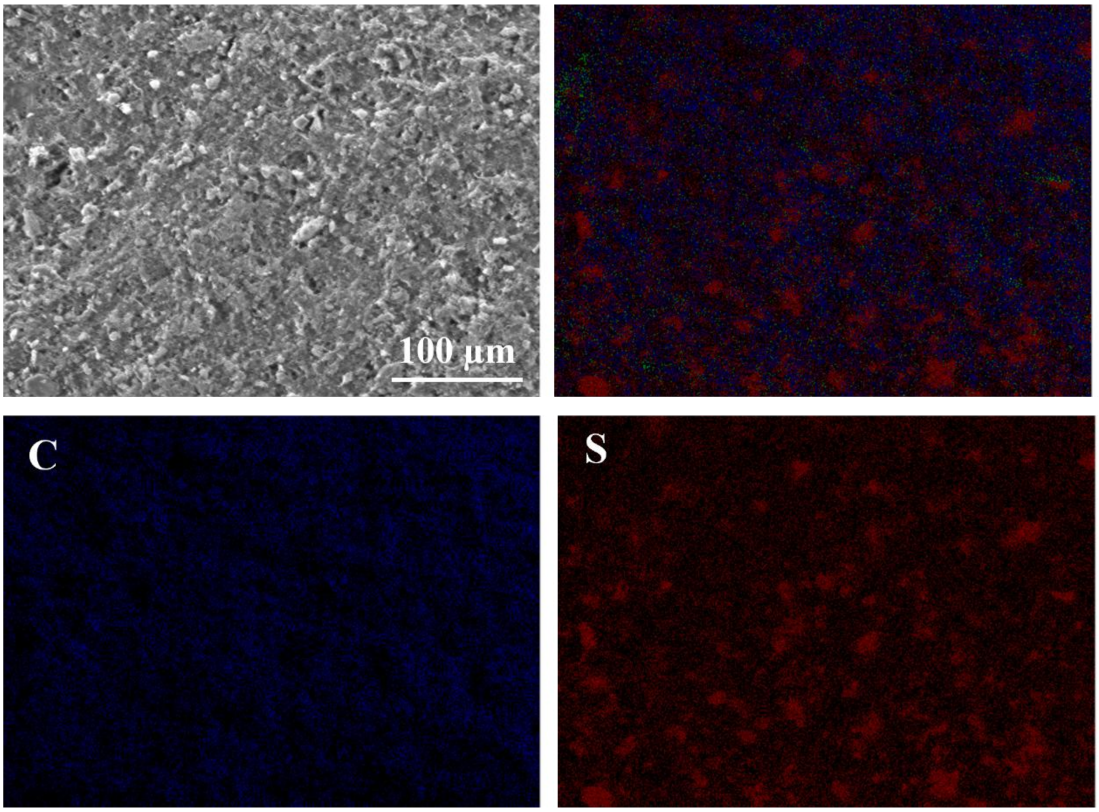

Fig. S1. EDS elemental mappings of TMTD-24S@ECP600JD.

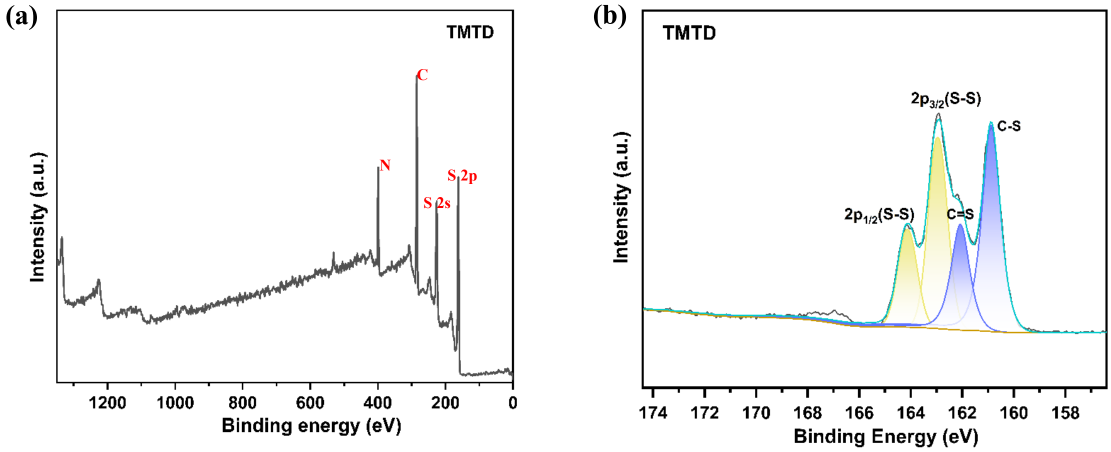

Fig.S2. (a) XPS full spectra of TMTD. (b) S 2p spectra.

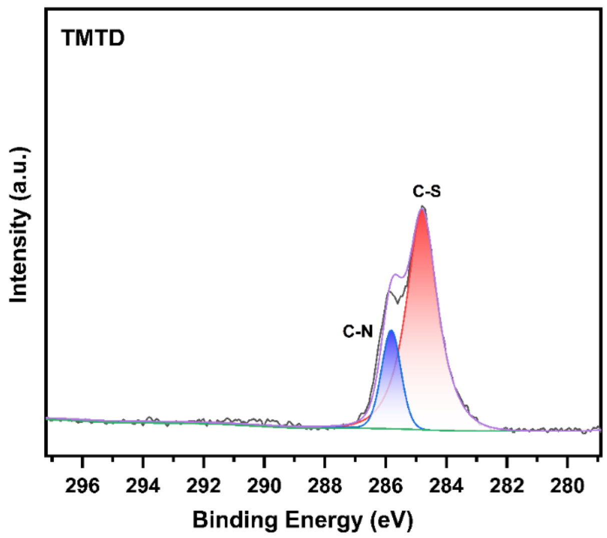

Fig.S3. C 1s spectra of TMTD.

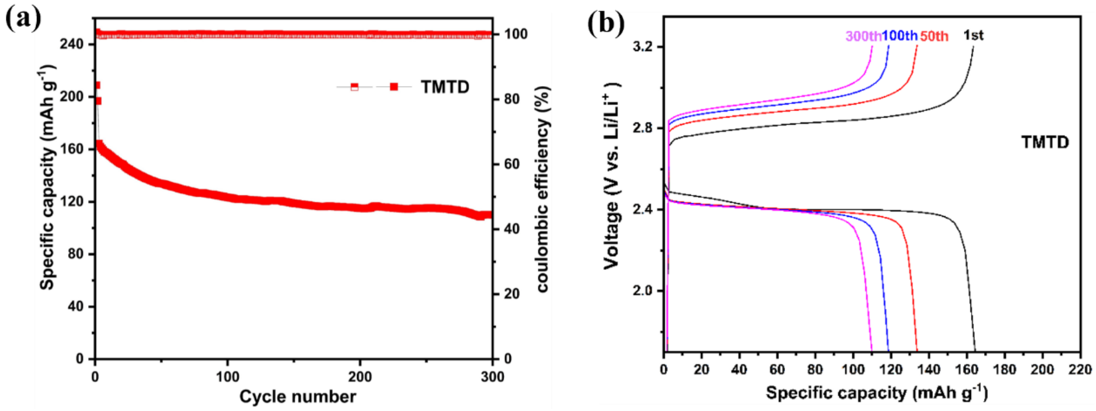

Fig.S4. (a) The cycling performance of TMTD cathode at 1C. (b) The charge and discharge curves at selected cycles.

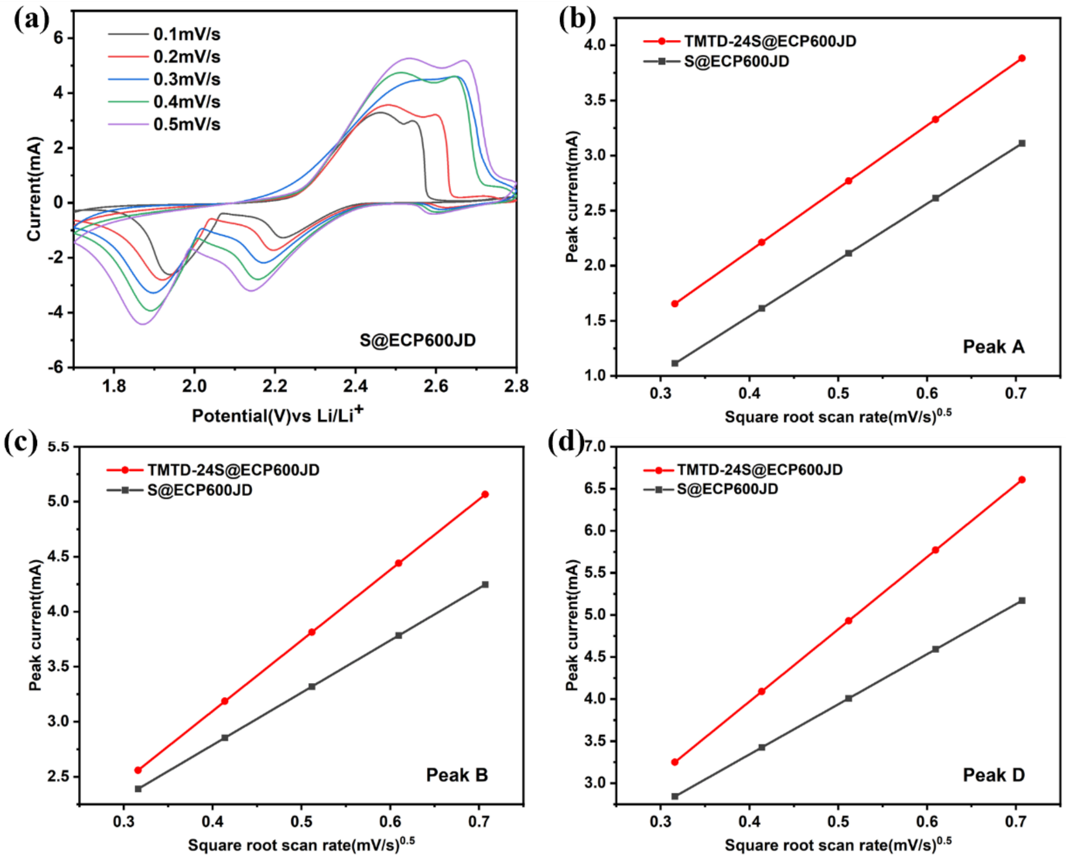

Fig.S5. (a) CV curves of S@ECP600JD cathode at scan rates from 0.1 to 0.5 mV s^{-1}. CV peak current of (b) peak A, (c) peak B and (d) peak D versus the square root of the scan rate.

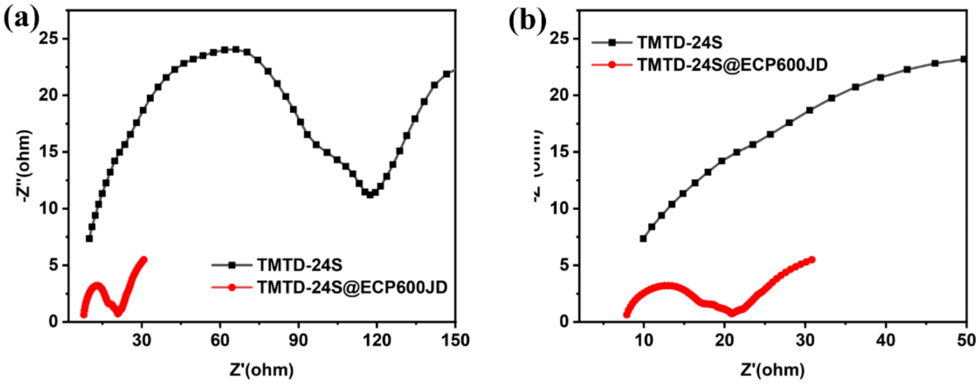

Fig.S6. EIS curves of the TMTD-24S and TMTD-24S@ECP600JD cathodes.

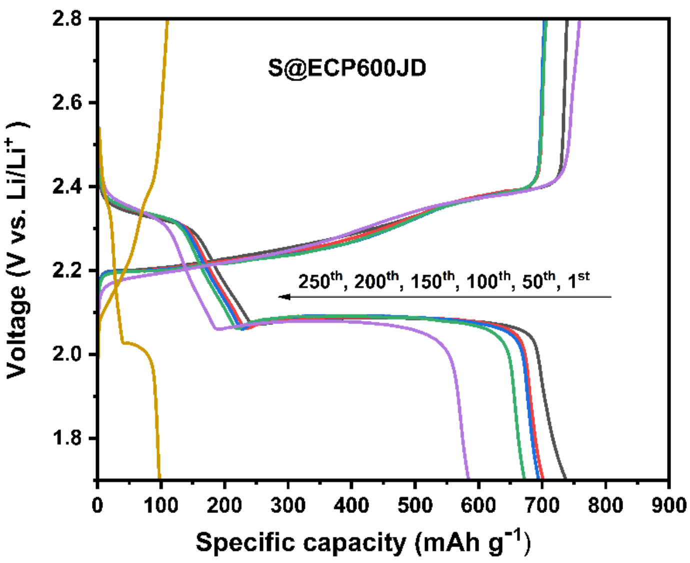

Fig.S7. Charge and discharge curves of S@ECP600JD cathode at selected cycles.

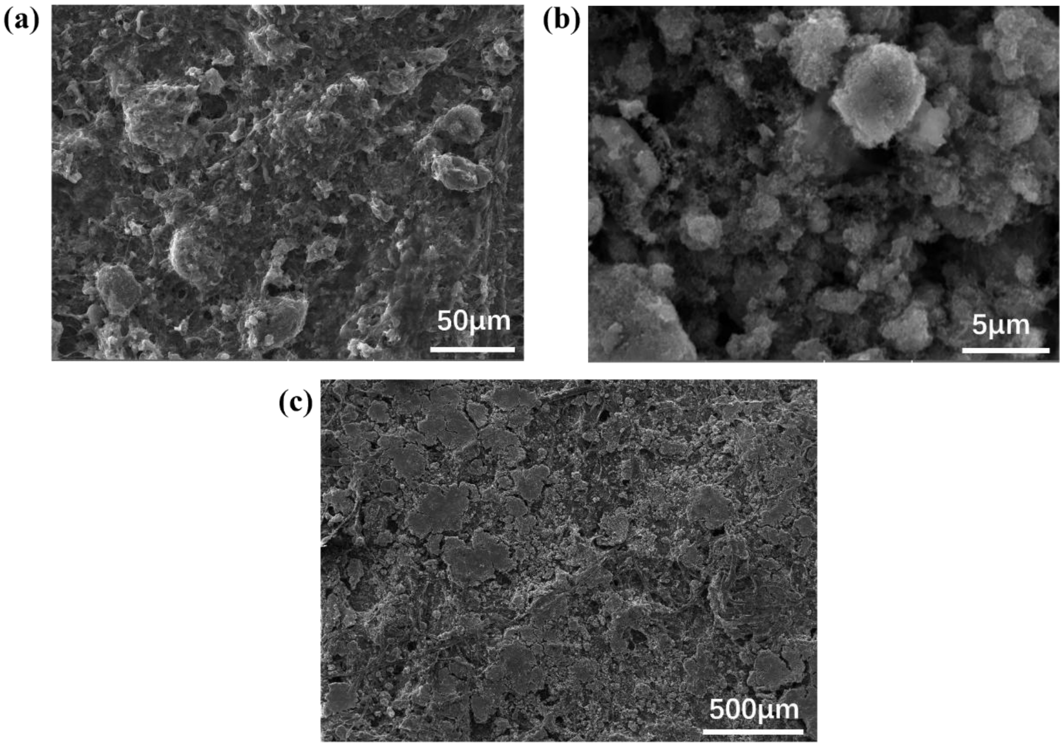

Fig.S8. SEM images of S@ECP600JD cathode: (a), (b) before cycling and (c) after cycling.

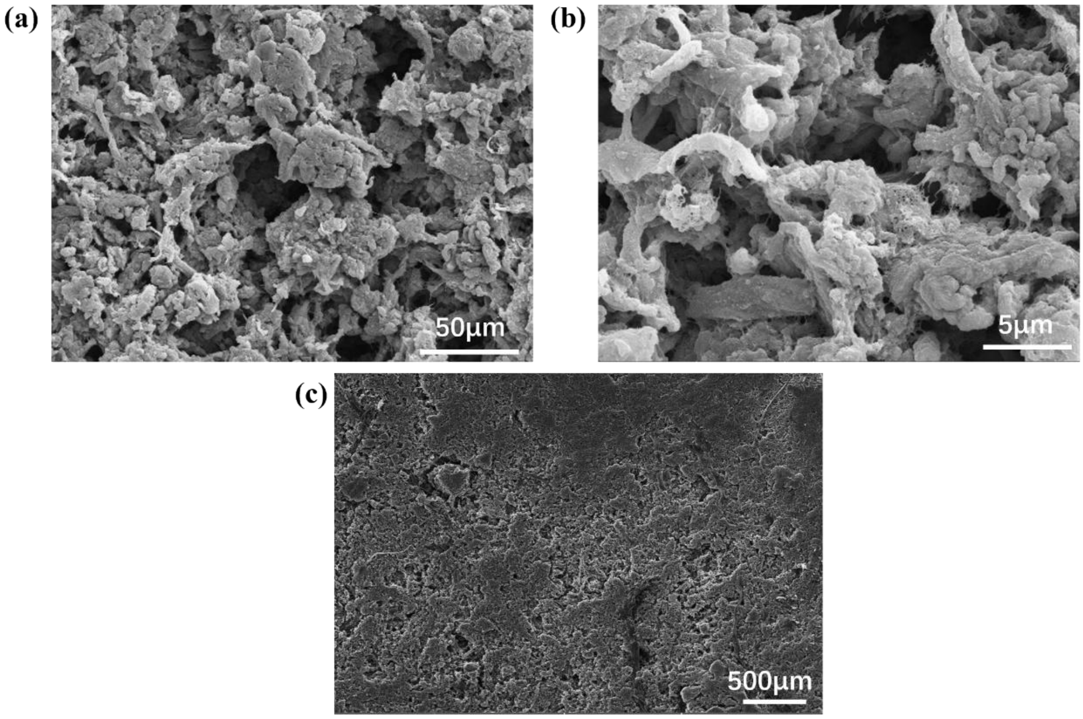

Fig.S9. SEM images of TMTD-24S@ECP600JD cathode: (a), (b) before cycling and (c) after cycling.

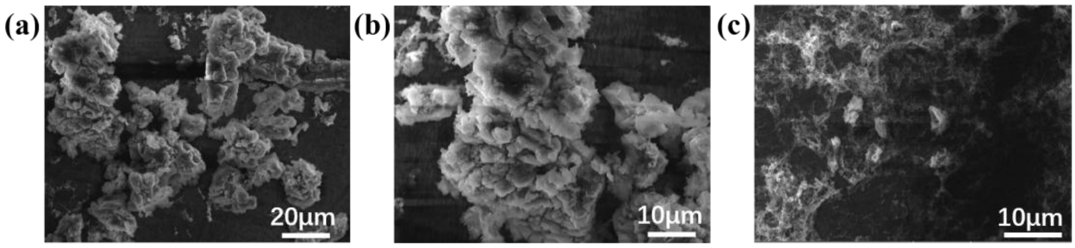

Fig.S10. SEM images of cycled separators from (a, b) S@ECP600JD//Li and (c) TMTD-24S@ECP600JD//Li cells

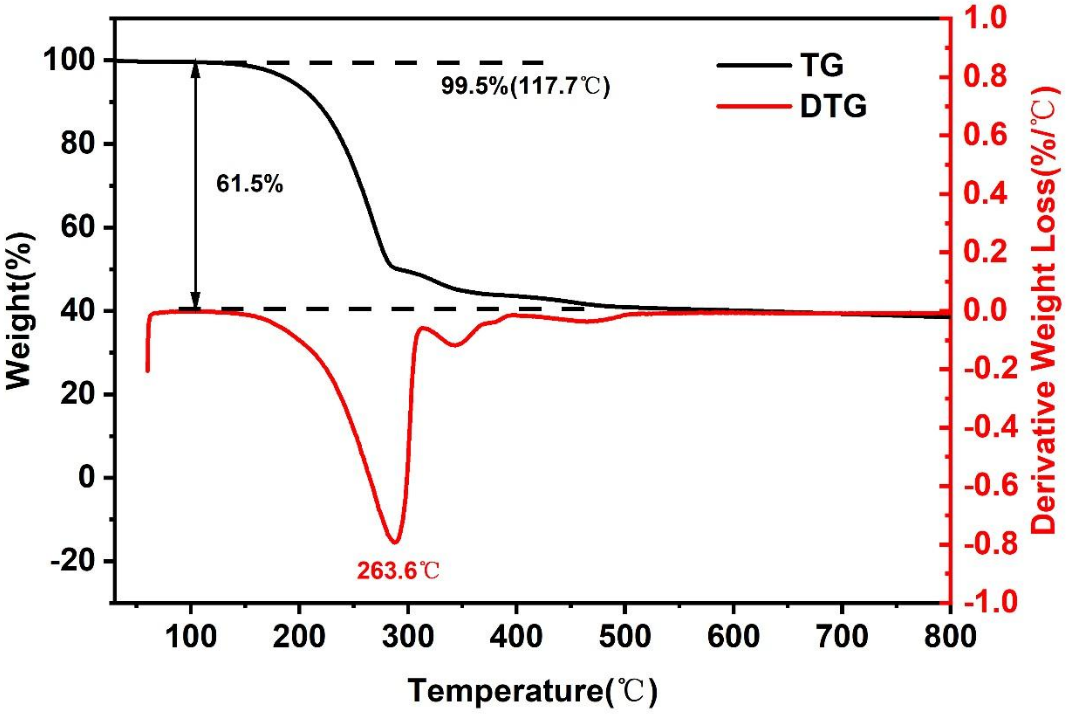

Fig. S11. TG-DTG curve of TMTD-54S@ECP600JD

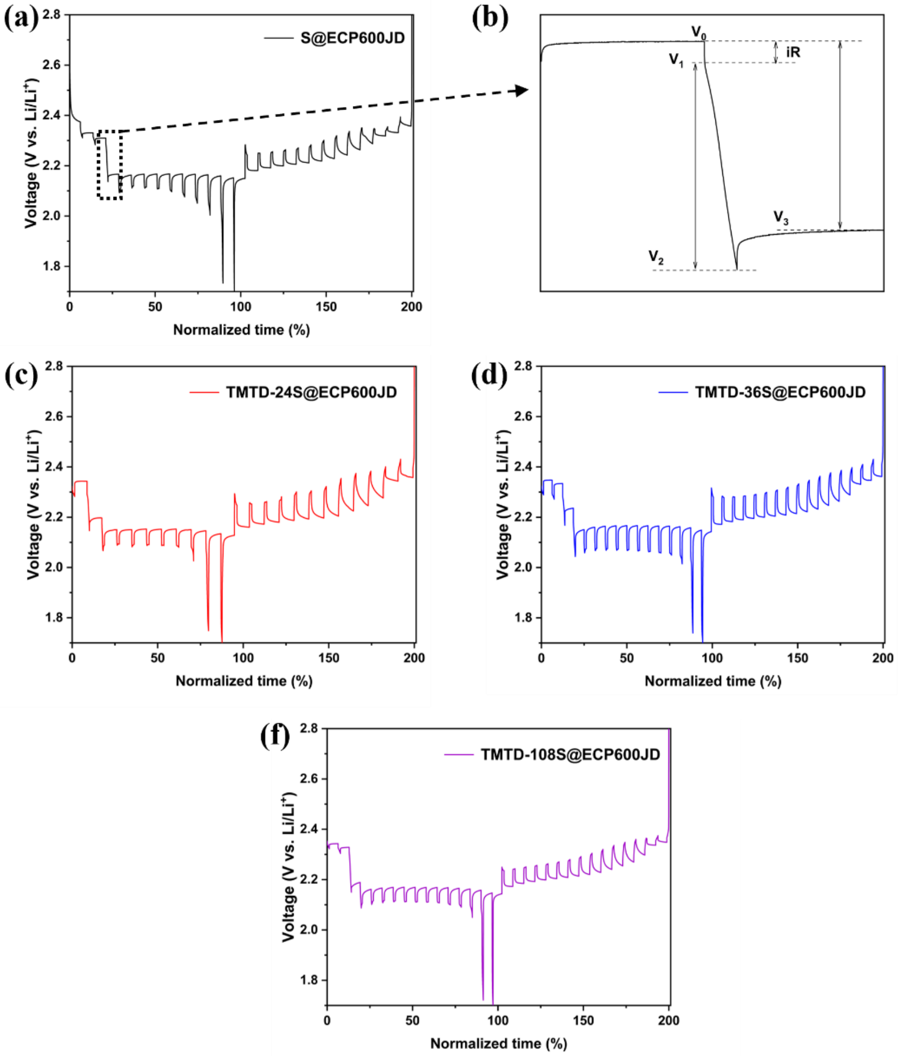

Fig.S12. GITT curves of TMTD-S@ECP600JD cathodes with different sulfur contents.

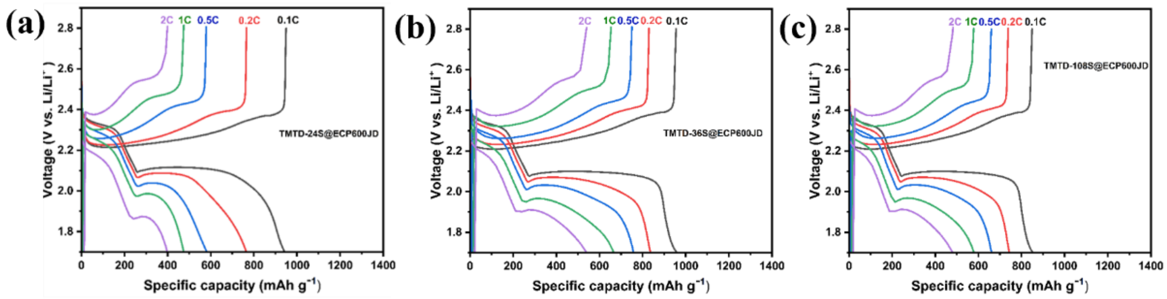

Fig.S13. Charge and discharge curves of (a)S@ECP600JD, (b) TMTD-36S@ECP600JD and (c)TMTD-108S@ECP600JD cathodes at different current densities.

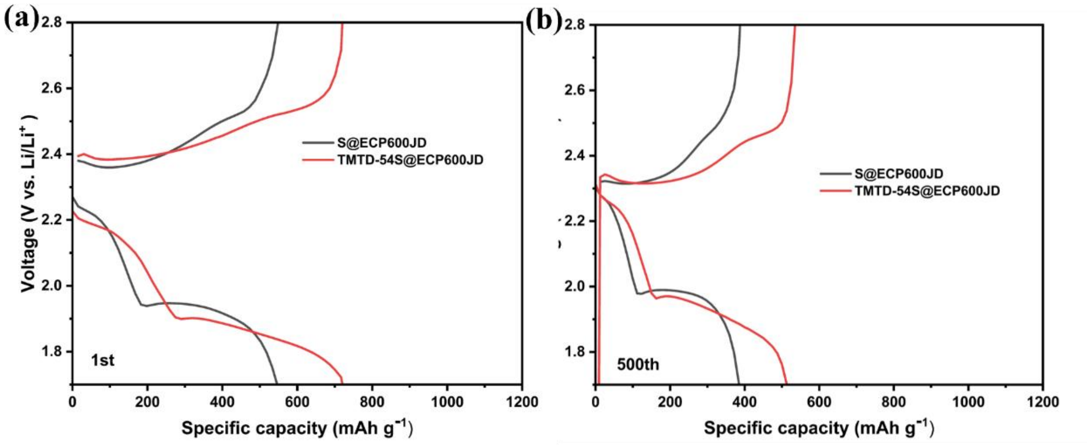

Fig.S14. Charge and discharge curves of (a) S@ECP600JD and (b) TMTD-54S@ECP600JD cathodes at the first and 500th cycles under 1C.

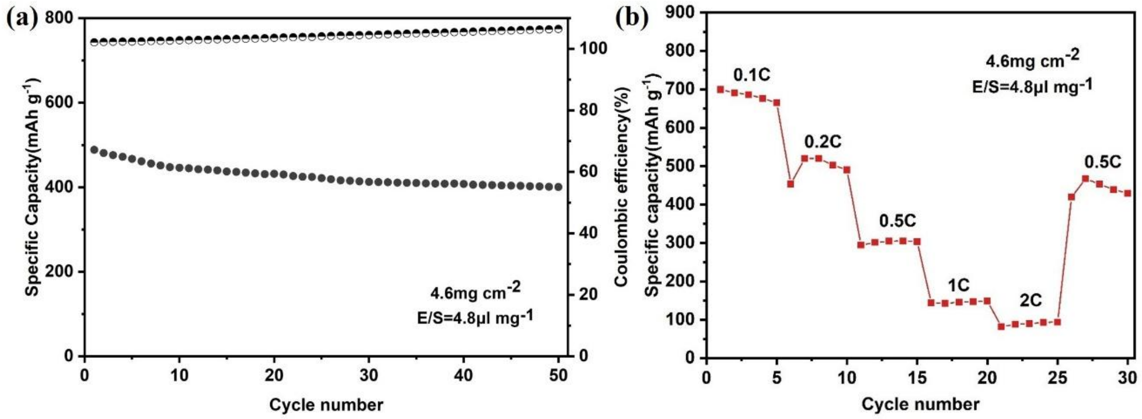

Fig.S15. (a) Cycling performance and (b) rate performance of TMTD-54S@ECP600JD cathode with high sulfur loading of 4.6 mg cm^{-2} and lean electrolyte of 4.8 μL mg^{-1}.

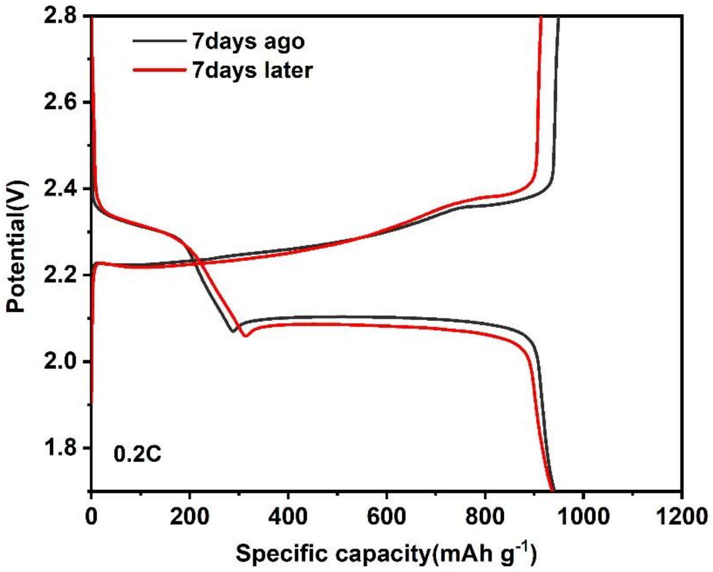

Fig.S16. The first charge and discharge curves of TMTD-54S@ECP600JD cathode before and after 7 days.

References

1. A. Gnanavel, D. Ovc-Okene, L. S. Shankar, L. Trif, R. Kun, J. Electroanal. Chem. 2025, 977, 118808.
2. J. Lei, J. Chen, H. Zhang, A. Naveed, J. Yang, Y. Nuli, J. Wang, ACS Appl. Mater. Interfaces 2020, 12, 33702.
3. A. Bhargav, A. Manthiram, Adv. Energy Mater. 2020, 10, 2001658.
4. A. A. Razzaq, X. Yuan, Y. Chen, J. Hu, Q. Mu, Y. Ma, X. Zhao, L. Miao, J.-H. Ahn, Y. Peng, Z. Deng, J. Mater. Chem. A 2020, 8, 1298.
5. S. Ma, Y. Wang, C. Fu, Y. Ma, Y. Gao, G. Yin, P. Zuo, Chem. Commun. 2020, 56, 12797.
6. Y. Zhao, Z. Quan, N. Xu, H. Zhang, Y. Chen, ACS Appl. Energy Mater. 2025, 8, 2360.
